# Targeting *PSEN1* by lnc-CYP3A43-2/miR-29b-2-5p to Reduce β Amyloid Plaque Formation and Improve Cognition Function

**DOI:** 10.3390/ijms231810554

**Published:** 2022-09-11

**Authors:** Wei Wuli, Shinn-Zong Lin, Shee-Ping Chen, Bakhos A. Tannous, Wen-Sheng Huang, Peng Yeong Woon, Yang-Chang Wu, Hsueh-Hui Yang, Yi-Cheng Chen, Renata Lopes Fleming, Jack T. Rogers, Catherine M. Cahill, Tsung-Jung Ho, Tzyy-Wen Chiou, Horng-Jyh Harn

**Affiliations:** 1 Bioinnovation Center, Buddhist Tzu Chi Medical Foundation, Hualien 97002, Taiwan; 2 Department of Life Science, National Dong Hwa University, Hualien 974301, Taiwan; 3 Department of Neurology, Hualien Tzu Chi Hospital, Hualien 97002, Taiwan; 4 Buddhist Tzu Chi Stem Cells Centre, Hualien Tzu Chi Hospital, Hualien 97002, Taiwan; 5 Neurochemistry Laboratory, Department of Psychiatry-Neuroscience, Massachusetts General Hospital and Harvard Medical School, 149 13th Street, Charlestown, Boston, MA 02129, USA; 6 Department of Nuclear Medicine, Taipei Medical University Hospital, Taipei 110301, Taiwan; 7 Taiwan Department of Molecular Biology and Human Genetics, Tzu Chi University, Hualien 97004, Taiwan; 8 Graduate Institute of Integrated Medicine, China Medical University, Taichung City 404, Taiwan; 9 Chinese Medicine Research and Development Center, China Medical University Hospital, Taichung City 404, Taiwan; 10 The Biotechnology Department, College of Medical and Health Science, Asia University, Taichung City 404, Taiwan; 11 Department of Medical Research, Hualien Tzu Chi Hospital, Hualien 97002, Taiwan; 12 Department of Medicine, Mackay Medical College, New Taipei City 25245, Taiwan; 13 Integration Center of Traditional Chinese and Modern Medicine, Hualien Tzu Chi Hospital, Hualien 97002, Taiwan; 14 Department of Chinese Medicine, Hualien Tzu Chi Hospital, Hualien 97002, Taiwan; 15 School of Post-Baccalaureate Chinese Medicine, Tzu Chi University, Hualien 97004, Taiwan; 16 Department of Pathology, Hualien Tzu Chi Hospital, Hualien 97002, Taiwan

**Keywords:** n-butylidenephthalide, Alzheimer’s disease, miR-29b-2-5p, Presenilin1, β-amyloid plaque

## Abstract

*Presenilin-1* (*PSEN1*) is a crucial subunit within the γ-secretase complex and regulates β-amyloid (Aβ) production. Accumulated evidence indicates that n-butylidenephthalide (BP) acts effectively to reduce Aβ levels in neuronal cells that are derived from trisomy 21 (Ts21) induced pluripotent stem cells (iPSCs). However, the mechanism underlying this effect remains unclear. This article aims to investigate the possible mechanisms through which BP ameliorates the development of Alzheimer’s disease (AD) and verify the effectiveness of BP through animal experiments. Results from RNA microarray analysis showed that BP treatment in Ts21 iPSC-derived neuronal cells reduced long noncoding RNA (lncRNA) CYP3A43-2 levels and increased microRNA (miR)-29b-2-5p levels. Bioinformatics tool prediction analysis, biotin-labeled miR-29b-2-5p pull-down assay, and dual-luciferase reporter assay confirmed a direct negative regulatory effect for miRNA29b-2-5p on lnc-RNA-CYP3A43-2 and *PSEN1*. Moreover, BP administration improved short-term memory and significantly reduced Aβ accumulation in the hippocampus and cortex of 3xTg-AD mice but failed in miR-29b-2-5p mutant mice generated by CRISP/Cas9 technology. In addition, analysis of brain samples from patients with AD showed a decrease in microRNA-29b-2-5p expression in the frontal cortex region. Our results provide evidence that the LncCYP3A43-2/miR29-2-5p/*PSEN1* network might be involved in the molecular mechanisms underlying BP-induced Aβ reduction.

## 1. Introduction

Approximately 6.2 million Americans aged 65 and more have Alzheimer’s disease (AD)-related dementia. The number is increasing at an alarming pace and so is the mortality caused by AD. Between 2000 and 2019, the number of deaths due to AD increased by 145% [1]. In this context, AD is the neuronal degeneration associated with β-amyloid (Aβ) plaques. Such plaques are composed of the amyloid peptide, and the 42-residue form β amyloid (Aβ1–42) is the predominant peptide causing AD [2]. Until now, amyloid-targeting therapies remain a prominent avenue for research as well [3]. The n-butylidenephthalide (BP) was initially extracted from the Chinese herbal medicine *Angelica sinensis* and can also be produced by chemical synthesis. BP can reduce Aβ deposits and the hyperphosphorylated status of Tau protein (p-tau) according to in vitro studies [4]. These results suggest that BP has the potential to treat AD. However, evidence of the BP effect on AD animal models is lacking. Thus, the efficacy of BP in reducing Aβ in AD is still worth exploring through in vivo studies.

Multiple genes play crucial roles in regulating Aβ produced in the brain. They include *amyloid precursor protein* (*APP*) [5], *beta-site APP-cleaving enzyme-1* (*BACE1*) [6], *presenilin-1* (*PSEN1*), and *presenilin-2* (*PSEN2*) [7]. Previous studies in the literature report that small molecule drugs can regulate γ-secretase by binding to *PSEN1*; which becomes a viable treatment strategy for treating AD [8]. In this study, we will discuss the effect of BP on genes associated with AD, and we consider *PSEN1* as the primary target. In recent years, several experiments demonstrated that aberrant miRNA expression is associated with Aβ production in AD [9,10]. For instance, loss of miR-29a/b-1 in sporadic AD correlates with increased *BACE1*/β-secretase expression and contributes to the increase of Aβ production [9]. Likewise, overexpression of miR-16 can cause decreased APP expression [10]. In this study, we analyze the PSEN1 binding affinity of BP through molecular docking calculation. In addition, we also explore other genes and miRNA that might be regulated by BP. In specific, the cell models of trisomy 21 (Ts21) induced pluripotent stem cells (iPSCs)-derived neurons [4] and human β-CTF/C6 cells [11] were used to investigate the mechanism of action of BP in the treatment of AD.

Furthermore, we used positron emission tomography-computed tomography (PET/CT) and Morris water maze to discuss the effect of BP on the behaviors of 3xTg-AD mice [12]. The Aβ deposition in the brain of 3xTg mice was detected by [18F]-Florbetaben PET/CT, and the spatial learning ability was tested by Morris water maze [13]. Moreover, we evaluated the association of miRNA-mRNA by establishing the miRNA mutant mice generated by CRISP/Cas9 technology and by analyzing the data collected using brain samples obtained from patients with AD [14]. Through these investigations, we aim to understand the potential of BP in treating AD in an in vivo setting and further explore the cellular and molecular mechanisms underlying BP’s effect in reducing Aβ levels.

## 2. Results

### 2.1. Decreased lnc-CYP3A43-2, PSEN1, and Aβ Production and Elevated miR-29b-2-5p Expression in BP-Treated Human Ts21-iPSC-Derived Neurons

**Figure 1:**
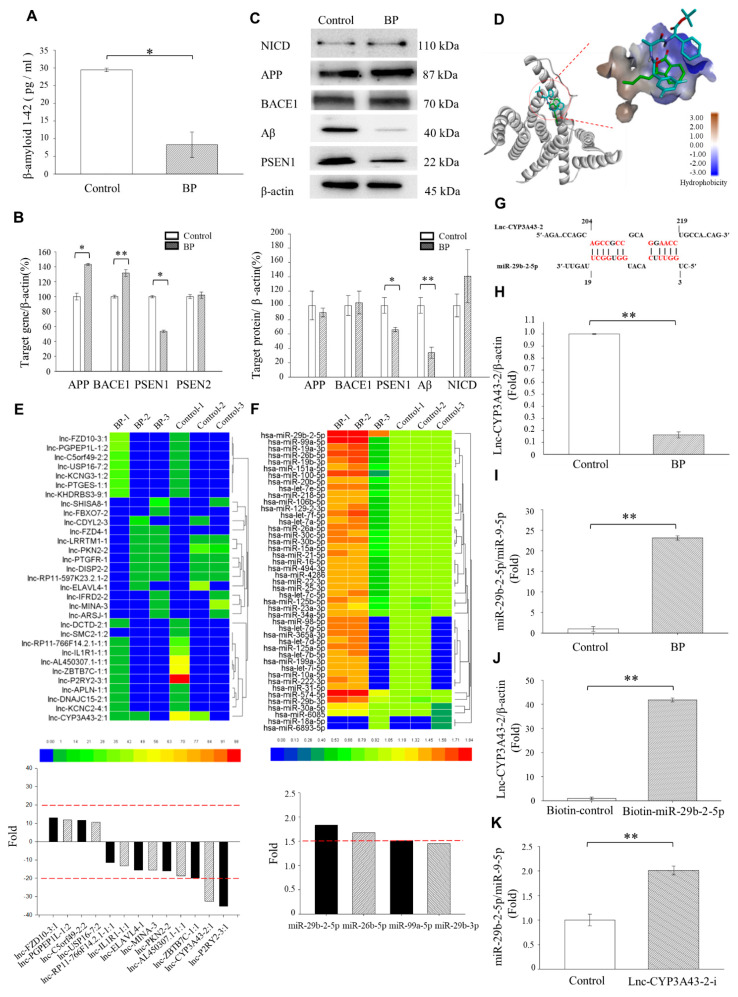
Increased miRNA-29b-2-5p expression and decreased *PSEN1* and Aβ expressions in BP-treated Ts21-iPSC-derived neurons. (**A**) Quantification of Aβ42 levels in the medium of control and BP-treated cells evaluated using ELISA (Control = 29.44 pg/mL ± 1.05, BP = 8.28 pg/ml ± 7.31), *n* = 3 for each group. (**B**) The expression of AD-associated gene/β-actin with or without 100 μM BP (*APP* of Control = 100% ± 4.5 vs. BP = 144.59% ± 1.19; *BACE1* of Control = 100% ± 1.79 vs. BP = 131% ± 4.44; *PSEN1* of Control = 100% ± 6.0 vs. BP = 51.9% ± 1.52), *n* = 3 for each group. (**C**) Representative western blot analysis of APP, BACE1, PSEN1, and Aβ levels after 48 h with or without 100 μM BP (*APP* of Control = 100% ± 24.03 vs. BP = 89.96% ± 7.54; BACE1 of Control = 100% ± 18.57 vs. BP = 103.94 % ± 20.79; PSEN1 of Control = 100% ± 16.37 vs. BP = 65.94% ± 6.86; Aβ of Control = 100% ± 16.13 vs. BP = 39.59% ± 6.63). (**D**) The binding structure simulation between BP (green stick) and DAPT (cyan stick) with PSEN1. (**Left**) The gray ribbon shows the 3D structure of PSEN1; (**right**) the enlargement of the active site of PSEN1 is shown by the surface model. The red color indicates the amino acids with strong hydrogen-bonding donor properties. (**E**,**F**) Gene microarray analysis of lncRNA and miRNA after BP treatment of Ts21-iPSCs and controls. (**G**) The predicted binding sites between lnc-CYP3A43 and miR-29b-2-5p. (**H**,**I**) RT-qPCR analysis of the expression levels of lnc-CYP3A43-2/β-actin and miR-29b-2-5p/miR-9-5p (lnc-CYP3A43-2/β-actin: Control = 1fold ± 0.02 vs. BP: 0.17fold ± 0.05; miR-29b-2-5p/miR-9-5p: Control = 1fold ± 0.09 vs. BP = 23.11fold ± 0.12), *n* = 3. (**J**) Real-time PCR results of miRNA expression from biotin-based pulldown assay validate the lnc-CYP3A43-2 targets of cellular miR-29b-2-5p (Control = 1fold ± 0.25 vs. BP = 41.81fold ± 0.09), *n* = 3. (**K**) Real-time PCR results of cellular miR-29b-2-5p expression levels after the inhibition of lnc-CYP3A43-2(Control = 1fold ± 0.17 vs. BP = 1.87fold ± 0.11), *n* = 3. * *p* < 0.05. ** *p* < 0.01.

We used Ts21-iPSCs-derived neurons for exploring the potential impact of BP treatment on Aβ production. The Ts21-iPSCs successfully differentiated into neurons and produced Aβ (Figure S1A–F). To test whether BP has a potential therapeutic effect on Ts21-iPSC-derived neurons, we first measured the amount of Aβ42 secreted in the culture medium in 100 μM BP-treated and untreated Ts21-iPSCs-induced neurons using ELISA. Approximately 5–10% of total Aβ exists as Aβ42; it has a considerably greater propensity for aggregation and is considered cytotoxic [15]. The results revealed a significant 3-fold decreased in Aβ42 production after BP treatment (Control = 29.44 pg/mL, BP = 8.28 pg/mL) (Figure 1A). Real-time polymerase chain reaction (PCR) was then conducted to analyze the effect of BP treatment on the major genes associated with AD. The expression levels of APP and BACE1 mRNAs were significantly elevated 30–40%, whereas *PSEN1* displayed about 40% decrease, but expression level of *PSEN2* was unaltered (Figure 1B). The mRNA expression of *APP*, *BACE1*, and *PSEN1* offers us a possible direction, but mRNA levels cannot be used as surrogates for corresponding protein levels [16,17]. Therefore, we also need to verify these results on the protein level. On the other hand, protein expression by western blot analysis detected about 30–40% decreased in PSEN1-carboxy-terminal fragments (CTF) protein levels. Although the mRNA expression of *APP* and *BACE1* genes increased after BP treatment of Ts21 neurons, at the protein level, APP and BACE1 were otherwise not significantly different in each group (Figure 1C). Moreover, consistent with the reduction in Aβ secreted in the culture medium, cytoplasmic Aβ accumulation in the Ts21-iPSC-derived neurons was reduced more than 60% by BP treatment. We concluded that BP treatment in Ts21-iPSC-derived neurons significantly reduced the Aβ protein expression and secretion (Figure 1C). As the Notch1 intracellular domain (NICD) is relevant to the development of AD drugs [18], we conducted western blot analysis to examine the effect of BP treatment on NICD levels. Our result showed that the expression of NICD protein was unchanged after BP treatment (Figure 1C).

Next, we used the AutoDock software to investigate whether BP binds directly to PSEN1/γ-secretase. We selected (N-[N-(3,5-difluorophenacetyl)-L-alanyl]-S-phenylglycine t-butyl ester (DAPT), a γ-secretase inhibitor, for positive control comparison. BP docked nicely into the active site of the PSEN1 protein (Figure 1D and Figure S2–S4). However, the predicted binding affinity of BP (−5.91 kcal/mol) was much weaker than DAPT (<−6 kcal/mol), hinting that BP has a relatively weaker affinity than DAPT to the active site of PSEN1 [19,20,21]. This observation prompted us to analyze the potential regulation of PSEN1 expression by miRNAs.

Long noncoding RNAs (lncRNAs) are non-protein coding RNA transcripts with more than 200 nucleotides. LncRNA functions as a competing endogenous RNA or miRNA sponge to sequester the miRNAs targeted the specific subsets of mRNA, thus preventing the miRNA-suppression mRNA [22]. The results of miRNA microarray on BP-treated Ts21-iPSCs-derived neurons revealed that the expression levels of two lncRNAs were greatly reduced (more than a 20-fold decrease) (Figure 1E), and the expression of six miRNAs was significantly increased (more than 1.5-fold increase) (Figure 1F). Using real-time PCR, we confirmed that the BP-treated Ts21-iPSC-derived neurons exhibited decreased lnc-CYP3A43-2 expression and elevated miR-29b-2-5p expression, where lnc-CYP3A43-2 level was reduced to 0.17-fold and miRNA-29b-2-5p level increased to 23.11-fold (Figure 1H,I). In conclusion, BP treatment effectively decreases Aβ production, while upregulating miR-29b-2-5p expression and downregulating lnc-CYP3A43-2, as well as PSEN1 mRNA and protein expression levels, in Ts21-iPSC-derived neurons.

### 2.2. lnc-CYP3A43-2 Interacts with miR-29b-2-5p

Using Bioinformatics tool to predict the binding energy between lncRNA and miRNA, we showed that the predicted binding energy between lnc-CYP3A43-2 and miR-29b-2-5p was the lowest among all other lnRNAs and miRNAs expressed in the brain (target candidates) (Table 1). Bioinformatics prediction analysis supported this calculation and showed the perfect binding sequence between lnc-CYP3A43-2 and miR-29b-2-5p (Figure 1G). We used a biotin-based pulldown assay to validate this interaction. A scrambled miRNA oligonucleotide sequence was used as a negative control. Lnc-CYP3A43-2 was successfully enriched in the pulldown products of miR-29b-2-5p compared to the control (Figure 1J). Inhibition of lnc-CYP3A43-2 increased the miR-29b-2-5p in Ts21-iPSC-derived neurons (Figure 1K). These results strongly suggested that lnc-CYP3A43-2 was a potential target of miR-29b-2-5p.

### 2.3. PSEN1 Is an miR-29b-2-5p Target Gene

**Figure 2:**
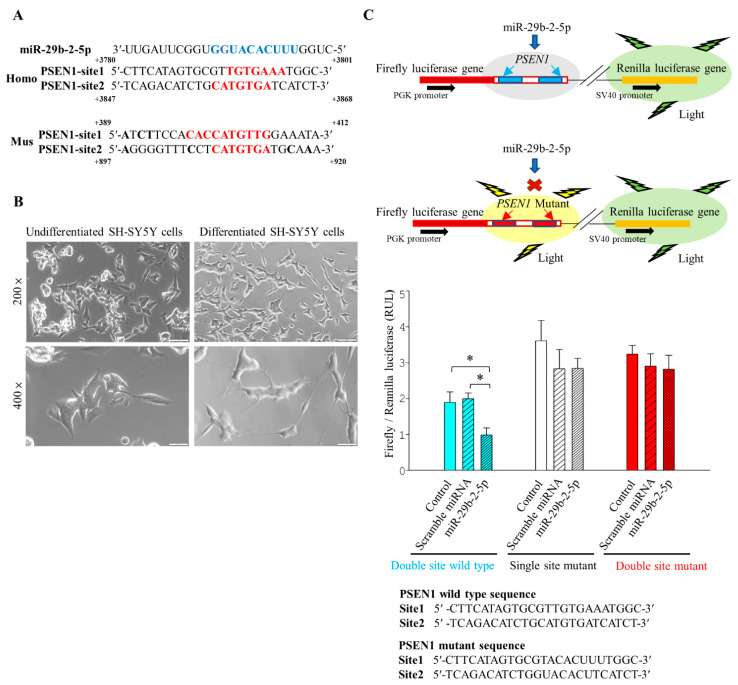
Luciferase assay indicated that miR-29b-2-5p targets 3′ UTR of *PSEN1* in SH-SY5Y cells. (**A**) Sequence and predicted base pairing of human miR-29b-2-5p with *PSEN1*. Two predicted target sites in human *PSEN1* 3′UTR are located at 3791–3797 and 3856–3862 nucleotides from the start of *PSEN1* 3′UTR. Mouse *PSEN1* 3′UTR are located at 397–404 and 908–914 nucleotides from the start of *PSEN1* 3′UTR. (**B**) Differentiated neuronal SH-SY5Y cells (**left**) are morphologically distinct from undifferentiated SH-SY5Y cells (**right**). Scale bars represent 100 μm (**up**) and 50 μm (**down**) (**C**). The pmirGLO vector is designed to quantitatively evaluate *PSEN1* activity by inserting *PSEN1* 3′UTR target sites downstream of the firefly luciferase gene, and the Renilla luciferase gene provides the necessary information normalization. The target sites of wild-type and mutant reporter constructs were transfected into neuronal SH-SY5Y cells alone or with 50 nM miR-29b-2-5p. The relative ratios of Renilla and firefly luciferase activity were measured. The expression of wild-type *PSEN1* decreased the expression of the reporter (blue bar). *PSEN1* single-site-2 mutation (white bar) or *PSEN1* double mutation (red bar) of miR-29b-2-5p abolished the inhibitory effect of miR-29b-2-5p on reporter expression The miR-29b-2-5p mimic significantly reduced the luciferase expression of the wild-type reporter (52.05%). This decrease in luciferase expression was diminished by mutation at one site in the reporter (74.31%) and abolished by two mutations in the reporter (86.97%). Blue bar: Control vs. Scramble miRNA vs. miR-29b-2-5p mimics = 1.89 RUL ± 0.29 vs. 1.99 RUL ± 0.16 vs. 0.98 RUL ± 0.19; White bar: Control vs. add scramble miRNA vs. add miR-29b-2-5p mimics = 3.60 RUL ± 0.56 vs. 2.82 RUL ± 0.53 vs. 2.68 RUL ± 0.63; Red bar: Control vs. add scramble miRNA vs. add miR-29b-2-5p mimics = 3.23 RUL ± 0.23 vs. 2.83 RUL ± 0.28 vs. 2.81 RUL ± 0.39. *n* = 3 for each group. * *p* < 0.05.

Bioinformatics tool prediction analysis revealed two potential putative miR-29b-2-5p target sites within the 3′ untranslated region (3′UTR) of human and mouse *PSEN1* genes (Figure 2A). The binding affinity of miR-29b-2-5p with 3′UTR of *PSEN1* of the first and second target sites are −12.5 kcal/mol and −14.7 kcal/mol (Table 2). The predicted binding affinity of miRNA-29b-2-5p with *PSEN1* was much stronger than that of BP with *PSEN1* (−5.91 kcal/mol).

We then generated a luciferase reporter construct containing the 3′UTR of wild-type or mutated target site on human *PSEN1* gene. To examine the influence of these complementary sites, we transfected this construct with the miR-29b-2-5p mimic into a neuroblastoma cell line (SH-SY5Y) (Figure 2B,C). The miR-29b-2-5p mimic significantly reduced the luciferase expression to 52.05% of the wild-type reporter. However, this decrease in luciferase expression was ameliorated (74.31%) by mutation at one site in the reporter and almost abolished (86.97%) by two mutations in the reporter (Figure 2C). These results confirmed that *PSEN1* is an miR-29b-2-5p target gene with two potential binding sites.

### 2.4. miR-29b-2-5p Decreases PSEN1 Expression and Aβ Formation

**Figure 3:**
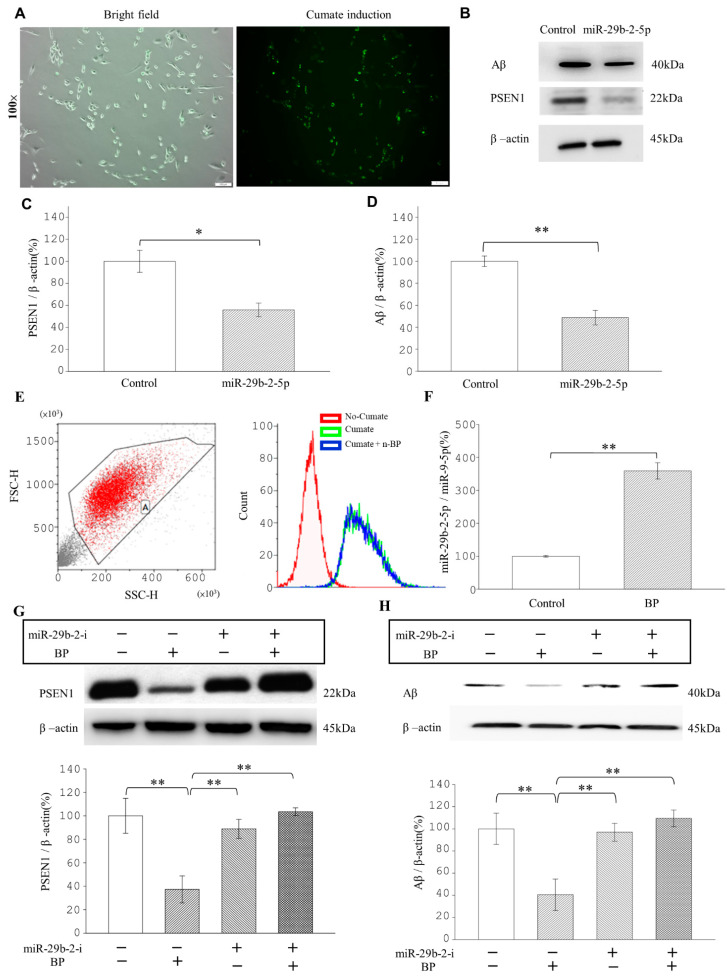
miRNA-29b-2-5p and BP decreased PSEN1 and Aβ expressions in β-CTF/C6 cells. (**A**) Left image, the morphology of β-CTF/C6 cells (bright field). Right image, green fluorescence emitted by β-CTF/C6 cells producing Aβ. Scale bars represent 100 μm. (**B**–**D**) miRNA-29b-2-5p treatment decreased PSEN1 protein expression and Aβ peptide levels. PSEN1: Control = 100% ± 15.45 vs. BP = 55.91% ± 15.25; Aβ: Control = 100% ± 8.01 vs. BP = 48.05% ± 0.11), *n* = 3 for each group. (**E**) Flow cytometry analysis of β-CTF/C6 cells treated with BP. Treat circled cells as a group (“A”) (**Left**). Right-Red peak, the fluorescence of β-CTF/C6 cells without Cumate activation (background). Green peak, with Cumate. Blue peak, with BP (**Right**). The similarity between the blue and green peaks indicates that BP does not affect the fluorescence of β-CTF/C6 cells. (**F**). RT-qPCR analysis of the expression levels of miR-29b-2-5p/miR-9-5p with or without 100 μM BP. miR-29b-2-5p/miR-9-5p: Control = 100% ± 3.74 vs. BP = 361.88% ± 7.44). Western blot analysis of PSEN1 (**G**) and Aβ (**H**) levels after 48 h with or without 100 μM BP in the absence or presence of miRNA-29b-2-5p. PSEN1/β-actin: Control = 100% ± 14.08 vs. BP = 41.45% ± 10.54 vs. miRNA-29b-2-i = 109% ± 3.44 vs. BP + miR-29b-2-i = 244.5% ± 11.15; Aβ/β-actin -Control = 100% ± 21.99 vs. BP = 40.81% ± 12.88 vs. miRNA-29b-2-i = 115% ± 9.8 vs. BP + miR-29b-2-i = 229.04% ± 12.85), *n* = 3 for each group. * *p* < 0.05. ** *p* < 0.01. miRNA-29b-2-i; miRNA-29b-2-inhibitor.

Next, we determined the relationship among miR-29b-2-5p, PSEN1, and Aβ. Because of the inefficiency of monitoring Aβ levels in Ts21-iPSC-derived neurons, it takes 30–50 days for Ts21 iPSCs to differentiate into nerves and produce Aβ. So, we conducted experiments using β-CTF/C6 cells. These are C6 glial cells transfected with β-CTF/C6 cells in a Cumate switch on/off system, which can produce Aβ and cause green fluorescence in activated cells [11] (Figure 3A). Western blot analysis revealed that miR-29b-2-5p significantly decreased 40–50% of the expressions of PSEN1 and Aβ (Figure 3B–D) compared to controls. Taken together, these results indicated that miR-29b-2-5p potentially decreased the expression of PSEN1 and the formation of Aβ by 40–50%.

### 2.5. BP Downregulates PSEN1 and Aβ Expression through miR-29b-2-5p

Next, we investigated the effect of BP treatment on the expression levels of miR-29b-2-5p, PSEN1, and Aβ in β-CTF/C6 cells. In β-CTF/C6 cells, the flow cytometry results demonstrated that 100 μM BP treatment did not affect Cumate expression (Figure 3E and Figure S5). Compared to the control group, the cell viability of β-CTF/C6 cells was significantly lower after adding 200 μM BP (71.1 %). Although 150 μM BP also reduced the cell viability in β-CTF/C6 cells (87.89 %), there was no statistically significant difference (*p*-value = 0.22) (Figure S6A).

The mRNA level of C99 in β-CTF/C6 cells is higher than that in original C6 cells. In addition, the Aβ levels increased in β-CTF/C6 cells after treatment with Cumate [11]. The result of cell viability by MTT assay showed that after a concentration of 600 μg/mL, Cumate induced β-CTF/C6 cells, and cell viability was significantly reduced (49.48%). Moreover, the protective effect of β-CTF/C6 cells was apparent at a BP concentration of 100 μM and 150 μM pretreatment but not at 50 μM. We observed that when pretreated with 50 μM BP plus 600 μg/mL Cumate, the cell viability decreased to 48.04%. However, when pretreated with 100 μM BP or 150 μM BP plus 600 μg/mL Cumate, the cell viability was restored to 89.95% and 66.32%, respectively (Figure S6B). The above results show that upon induction with 600 μg/mL, pretreatment with BP 100 and 150 μM increased cell viability and decreased cell toxicity from Aβ formation, but this effect was not observed with 50 μM BP pretreatment.

The BP-treated β-CTF/C6 cells exhibited 3.5-fold elevation in miR-29b-2-5p expression (Figure 3F) and a reduction in PSEN1 and Aβ expressions to 41.45% and 40.81%, respectively (Figure 3G,H). To examine whether BP inhibited PSEN1 and Aβ expression through miR-29b-2-5p, we transfected the miR-29b-2-5p inhibitor into β-CTF/C6 cells and analyzed the inhibitory effect of BP on PSEN1 and Aβ expression. Results showed that adding the miR-29b-2-5p inhibitor into β-CTF/C6 cells abolished the decrease in PSEN1 and Aβ expression induced by BP (Figure 3G,H). These findings indicated that BP treatment decreased the protein expression of PSEN1 and Aβ by increasing the expression of miR-29b-2-5p.

### 2.6. BP Upregulates miRNA-29b-2-5p and Downregulates PSEN1 in 3xTg AD Mice Model

**Figure 4:**
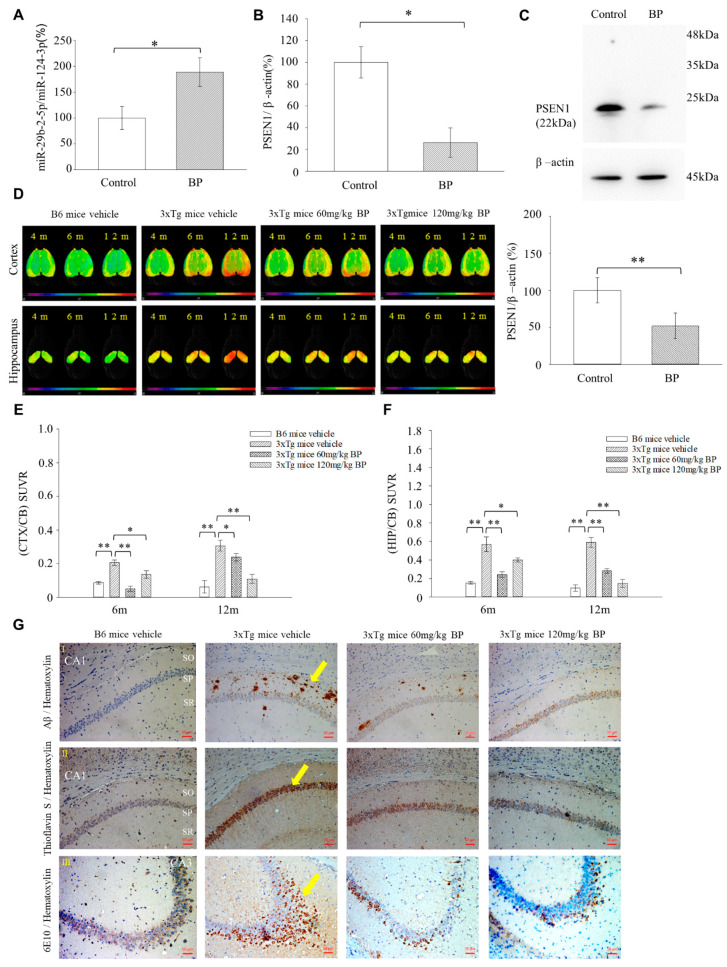
BP decreased Aβ deposition in the hippocampus of 3xTg mice. RT-PCR analysis of the expression levels of miR-29b-2-5p gene (**A**) and *PSEN1* gene (**B**) in the hippocampus of BP-treated 3xTg mice (miR-29b-2-5p: Control = 100% ± 28.4 vs. BP = 189.49% ± 14.82; *PSEN1*: Control = 100% ± 19.22 vs. BP = 26.26% ± 23.24), *n* = 4. (**C**) western blot analysis of PSEN1 in the hippocampus of BP-treated 3xTg AD mice. PSEN1 was reduced in the BP treated group (Control = 100% ± 23.20 vs. BP = 51.79% ± 19.73), *n* = 4. * *p* < 0.05. ** *p* < 0.01. (**D**) 3D radiotracer [18F]-FBB images show Aβ accumulation in the brains of 3xTg mice. After 4–12 months, the B6 control mice exhibited no Aβ accumulation (green color). The 3xTg transgenic mice demonstrated amyloid deposits in the hippocampus (HIP) and cortex (CTX) of the brain and tended to accumulate amyloids after 12 months of birth (orange-red color). Oral administration of BP (60 or 120 mg/kg) resulted in low levels of amyloid accumulation. SUVR; standard uptake value ratio, M; months, cerebellum: CB. Comparison of VOI-based [18F]-FBB SUVR (CTX/CB) (**E**) and SUVR (HIP/CB) (**F**) between vehicle-treated B6 mice, vehicle-treated 3xTg transgenic, and BP-treated 3xTg mice (60 mg/kg and 120 mg/kg) at the age of 6 and 12 months (CTX/CB-6m: Vehicle-treated B6 mice = 0.09 ± 0.01, Vehicle-treated 3xTg mice = 0.21 ± 0.04, 60 mg/kg BP-treated 3xTg mice = 0.06 ± 0.01, 120 mg/kg BP-treated 3xTg mice = 0.13 ± 0.05; CTX/CB-12m: Vehicle-treated B6 mice = 0.06 ± 0.04, Vehicle-treated 3xTg mice = 0.30 ± 0.03, 60 mg/kg BP-treated 3xTg mice = 0.23 ± 0.03, 120 mg/kg BP-treated 3xTg mice = 0.10 ± 0.03; HIP/CB-6m: Vehicle-treated B6 mice = 0.15 ± 0.02, Vehicle-treated 3xTg mice = 0.56 ± 0.16, 60 mg/kg-treated 3xTg mice = 0.24 ± 0.05, 120 mg/kg-treated 3xTg mice = 0.39 ± 0.04; HIP/CB-12m: Vehicle-treated B6 mice = 0.09 ± 0.07, Vehicle-treated 3xTg mice = 0.59 ± 0.11, 60 mg/kg-treated 3xTg mice = 0.28 ± 0.04, 120 mg/kg-treated 3xTg mice = 0.14 ± 0.08), *n* = 4 for vehicle-treated and *n* = 5 for vehicle-treated 3xTg mice, 60 mg/kg BP-treated 3xTg mice and 120 mg/kg BP-treated 3xTg mice. (**G-I**) Immunostaining with Aβ antibody to detect amyloid deposition in the CA1 area of the hippocampus. Brown dots indicated Aβ plaque accumulation (Yellow arrow). We observed Aβ plaques on both sides of the sp in vehicle-treated 3xTg transgenic mice but not in vehicle-treated B6 mice. Moreover, the Aβ plaques of 60 and 120 mg/kg BP-treated 3xTg mice were significantly reduced. (**G-II**) Results of Th-S staining. A similar trend of Aβ plaques in the SP areas of each group was observed in the SP area of CA1 using Th-S staining (Yellow arrow). Brain sections were selected at −2.2 mm posterior to the bregma. *n* = 4 for B6 mice vehicle, vehicle-treated 3xTg mice *n* = 5 for 60 mg/kg and 120 mg/kg BP-treated 3xTg mice. (**G-III**) Immunostaining with 6E10 antibody to detect amyloid deposition in the hippocampus. Brown dots indicated amino acids 6–10 of Aβ accumulation (Yellow arrow). We observed amino acids 6–10 of Aβ from CA1 diffuse to CA3 in vehicle-treated 3xTg transgenic mice but not in B6 mice. Moreover, the amino acids 6–10 of Aβ of 60 and 120 mg/kg BP-treated 3xTg mice were significantly reduced. *n* = 4 for B6 mice vehicle, vehicle-treated 3xTg mice. *n* = 5 for 60 mg/kg and 120 mg/kg BP-treated 3xTg mice. * *p* < 0.05. ** *p* < 0.01. Scale bars represent 50 μm. SO: stratum oriens; SP: stratum piramidale; SR: stratum radiatum. CA: Cornu Ammonis areas.

Next, we validated the regulation of miR-29b-2-5p and *PSEN1* in the brain by BP using 3xTg AD mice. The BP treatment of 3xTg AD mice increased the expression of miR-29b-2-5p by almost 2-fold and decreased the expression of *PSEN1* by more than 66% in the hippocampus (Figure 4A,B). Western blot analysis of PSEN1 in the hippocampus under the 120 mg/kg BP treatment showed a 48% reduction in PSEN1 (Figure 4C). These results showed the consistency of miR-29b-2-5p in downregulating PSEN1 after BP treatment in both 3xTg AD mice and β-CTF/C6 cells (Figure 3B,C,F).

### 2.7. BP Reduces Aβ Plaque Formation in the Hippocampus and Cortex of Triple Transgenic (3xTg) AD Mice

We first investigated the effect of BP treatment on Aβ accumulation in the hippocampus and cortex of mice at the age of 4–12 months by [18F]-Florbetaben (FBB) positron emission tomography-computed tomography (PET-CT) (Figure 4D,F, Figures S7 and S8). Our results demonstrated that non-transgenic (C57BL/6, B6) mice exhibited almost no Aβ accumulation in the hippocampus or cortex. However, in 3xTg AD mice, Aβ accumulation in the hippocampus and cortex increased with age (Figure 4D). Treatment with BP decreased Aβ accumulation in the whole brains of 6- and 12-month-old 3xTg AD mice (Figure 4E,F and Figure S8). The standardized uptake value ratio (SUVR) of FBB in the cortex and hippocampus was significantly lower at age twelve months than at age six months when the mice were treated with 120 mg BP/kg body weight. The SUVR 18F-FBB uptake by 6-month-old 3xTg mice in the cortex versus vehicles were 0.21 and 0.13, respectively; whereas SUVR uptake in 12-month 3xTg mice were 0.30 and 0.10, respectively. The uptake levels retained in the hippocampus were 0.56 and 0.39, respectively (6-month-old), and 0.59 and 0.14 (12-month-old), respectively. In addition, 3xTg mice developed Tau hyperphosphorylation in the brain after nine months of birth, which can be detected at the threonine (position 181 [p-Tau181]) through [18F]-THK5351. In this context, hyperphosphorylation of Tau protein in neurons is one of the causes of Alzheimer’s disease [23]. Our experimental results showed that p-Tau181 in the cortex decreased in 3xTg mice after administration BP at doses of 60 mg/kg and 120 mg/kg, but there was no statistically significant difference (Figure S9). The hippocampus is the most relevant part responsible for memory in the brain. Therefore, we examined Aβ levels in the hippocampus of 14-month-old 3xTg AD mice and performed immunostaining Aβ plaques by Aβ antibody and Thioflavin S (Th-S). We observed that Aβ production was absent in the CA1 area of the hippocampus of B6 mice but present in that of 3xTg AD mice. Compared to 3xTg AD mice treated with the vehicle, the 3xTg AD mice treated with 120 mg/kg BP displayed significantly reduced Aβ production in the CA1 area of the hippocampus (Figure 4G). The APP/Aβ reactive amino acid residue 1–17 of beta-amyloid can be detected using monoclonal antibody 6E10 staining in the CA3 area of the hippocampus of 3xTg AD mice [24,25]. The results obtained from Th-S and Aβ antibody were consistent with the immunostaining results by the 6E10 antibody (Figure 4G). In addition, we checked the protein expression levels by western blot. Indeed, 120 mg/kg BP-treated 3xTg AD mice exhibited significantly reduced Aβ protein expression levels in the brain (Figure S10). These results strongly suggested that BP potentially reduced amyloid deposition in the mouse hippocampus and cortex brain regions.

### 2.8. BP Ameliorates Spatial Learning and Memory Deficits in Aged 3xTg AD Mice

**Figure 5:**
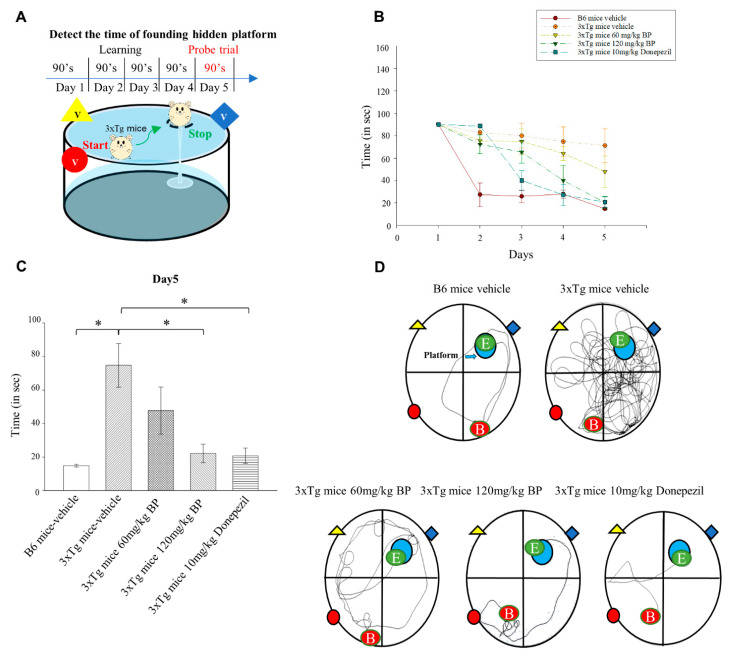
BP ameliorates spatial learning and memory in 3xTg AD mice. (**A**) We have trained the B6 mice and 3xTg mice to find the platforms hidden in the water. Our training time is five days, 90 s each time, once a day. s: second (**B**) The time spent to reach the platform in each group on Day 1 to Day 5. (**C**) Time spent in the target quadrant during the test for each group on Day 5. Time spent in the target: B6 mice—vehicle = 14.86 s ± 1.49, 3xTg mice—vehicle = 74.74 s ± 23.83, *n* = 6; 60 mg/kg BP-treated 3xTg mice = 47.84 s ± 25.69, *n* = 4; 120 mg/kg BP-treated 3xTg mice = 22.22 s ± 10.14, *n* = 6; 10 mg/kg treated 3xTg mice = 20.83 s ± 8, *n* = 4. * *p* < 0.05. (**D**) Representative swimming route map of each group on the last day of training. Red dot, the starting point of the swim; green dot, the end point of the swim. The marker of the red circle, yellow triangle, and blue diamond are visual cues.

To examine whether BP can improve spatial learning and memory in aged 3xTg AD mice, we conducted the Morris water maze test on 3xTg AD mice at nine months of age (Figure 5A). The 3xTg AD mice treated with the vehicle lacked spatial learning and memory ability, as demonstrated by no decrease in the time needed to reach the platform from Day 2 to Day 5 (Figure 5B). Donepezil is an approved clinical therapy for Alzheimer’s, the results recorded on Day 5 showed that 120 mg BP/kg exhibited similar effects as those of 10 mg Donepezil/kg on spatial learning and memory (Figure 5C,D) reducing more than 60% of the time required in the Morris water maze test. These data suggest that BP treatment ameliorates the impairment in spatial learning and memory in 3xTg AD mice. The toxicity studies on 3xTg mice treated with oral 60 or 120 mg/kg BP and 10 mg/kg Donepezil did not significantly alter the survival periods of these mice (Figure S11).

### 2.9. Mutation of miR-29b-2-5p Resulted in the Upregulation of PSEN1 and Aβ Expression in the Gene-Edited Mouse Model

**Figure 6:**
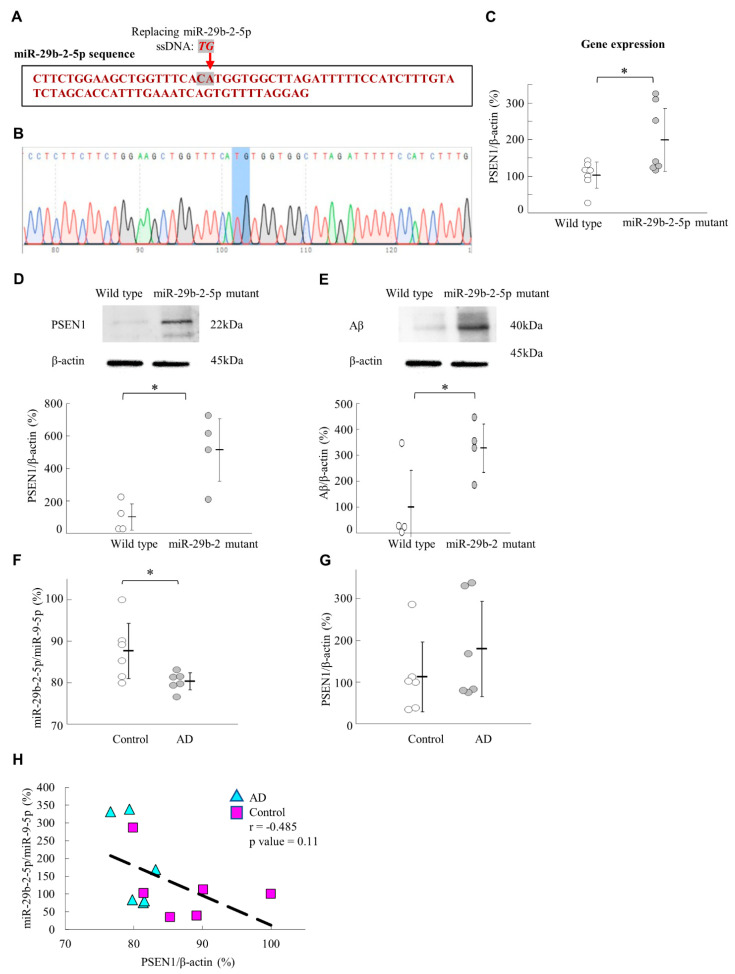
miRNA-29b-2-5p negatively regulates *PSEN1* expression and Aβ. (**A**) The red-labeled nucleotides are wild-type miR-29b-2-5p sequences. In the mutant mice, CA nucleotides were replaced with TG. (**B**) The DNA sequence mapping of miR-29b-2-5p in mir29b-2-5p mutant mice. The C change to T and A change to G. (**C**) RT-qPCR analysis of the expression levels of *PSEN1* in the hippocampus of miR-29b-2-5p-mutant mice (Wild type = 100% ± 36.86 vs. miR-29b-2-5p mutant = 193.45% ± 46.80), *n* = 7. (**D**,**E**) western blot analysis of PSEN1 and Aβ in the hippocampus of wild type mice and miR-29b-2-5p-mutant mice (PSEN1/β-actin: Wild type = 100% ± 92.92 vs. miR-29b-2-5p mutant = 515.24% ± 43.25; Aβ/β-actin: Wild type = 100% ± 164.93 vs. miR-29b-2-5p mutant = 341.97% ± 31.85), *n* = 4 for each group. The expression levels of miR-29b-2-5p (**F**) and *PSEN1* (**G**) in human brain BA9 specimens compared with those of control groups using RT-qPCR analysis. *n* = 6 for each group. (miR-29b-2-5p/ miR-9b-5p: Control = 100% ± 8.62 vs. AD = 91.61% ± 2.81; PSEN1/β-actin: Control = 100% ± 81.52 vs. AD = 159.87% ± 69.66) (*n* = 6 for each group), * *p* < 0.05. (**H**) The correlation analysis between miR-29b-2-5p and *PSEN1* in human brain BA9 specimens; correlation coefficient (r) = −0.485, *p* = 0.11. The average age of control vs. AD = 84.3 ± 2.9 vs. 85.3 ± 3.4; The average post-mortem interval of control vs. AD = 420 min ± 346 vs. 375 min ± 252.

We developed miR-29b-2-5p mutant mice to further confirm the relationship between miR-29b-2-5p and PSEN1. Figure 6A depicted the experimental design of the pronuclear injection of miR29b-2 gene-edited mice generated by CRISP/Cas9. The CA nucleotides were replaced with TG in the miR-29b-2-5p sequence (Figure 6B). We detected increased PSEN1 expression in the miR-29b-2-5p mutant mice. In addition, the gene expression levels of PSEN1 in the hippocampus of miR-29b-2-5p-mutant mice were upregulated to 193.45% as compared to wild type mice (Figure 6C). Western blot analysis of PSEN1 and Aβ in the hippocampus of wild type mice and miR-29b-2-5p-mutant mice validated the negative relationship between miR-29b-2-5p and *PSEN1*, where PSEN1 and Aβ expression levels were upregulated to 515.24% and 341.97% in the hippocampus of miR-29b-2-5p-mutant mice, respectively (Figure 6D,E).

### 2.10. miR-29b-2-5p Is Dysregulated in the Brain of Patients with AD

In patients AD, the frontal cortex (BA9) revealed Aβ deposition [26], and previous studies showed that BA9 tissue samples are suitable for detecting miRNA and the associated genes that play a role in Alzheimer’s disease [27,28]. In this study, the levels of miR-29b-2-5p were significantly lower in patients with AD than those in controls (Control vs. AD = 100% vs. 91.61%) (Figure 6F). On the other hand, the expression level of *PSEN1* in patients with AD was higher than that in controls (Control vs. AD =100 % vs. 159.87%) (Figure 6G). However, correlation analysis did not support the correlation model between *PSEN1* and miR-29b-2-5p expressions (*p* = 0.11) (Figure 6H). In the miR-29b family, the pre-miR-29b-2 hairpin is cleaved by the RNase III enzyme Dicer to generate miR-29b-2-5p and miR-29b-3p [29]. In this context, we also discovered that the expression levels of miR-29b-3p were not affected in the BA9 samples of the human brain between AD specimens and age-matched controls (Figure S12).

## 3. Discussion

This study aimed to determine the molecular mechanism underlying the action of BP in AD. In vitro results indicated that the interaction between lnc-CYP3A43-2 and miR-29b-2-5p is implicated directly in Aβ formation. We also identified *PSEN1*, which is involved in Aβ production, as a target of miR-29b-2-5p, both in vitro and in vivo. We further validated the negative correlation between miR-29b-2-5p and *PSEN1* expression in clinical human AD samples. We realized that a further, more extensive sample analysis is helpful in solidifying our hypothesis. Nevertheless, these results concisely emphasized the importance of the lncRNA (LncCYP3A43-2)-miRNA(miR29-2-5p)-mRNA (*PSEN1*) network in AD pathogenesis.

An early intervention strategy for AD treatment was to inhibit the cleavage of γ-secretase and reduce the production of Aβ [15]. Recent research focused on investigating the use of γ-secretase modulators (GSMs) as AD treatments [30]. In fact, γ-Secretase contains PSEN1, Nicastrin, Anterior pharynx-defective 1, and Presenilin enhancer 2.

Increasing evidence indicates that targeting *PSEN1* regulation reduced the production of β-amyloid, the main component for Aβ plaque, without affecting the Notch signaling pathway. Besides, it does not affect NICD formation [18,31]. Our data demonstrated that BP treatment did not affect NICD production. We speculated that the action of BP is through regulating LncCYP3A43-2/miR-29b-2-5p and in turn downregulating *PSEN1* expression rather than inhibiting all four components of γ-secretase. This explained why Notch signaling was not affected by BP treatment. Further in-depth studies are needed before forming a complete picture of BP effects on improving AD.

The Ts21-neuron has *APP* and *SOD1* gene alterations; the model displayed Aβ production from APP source and intermediate processes in neurons. Our study disclosed that BP did not inhibit APP directly but rather worked via reducing *PSEN1* expression in Ts-21 neurons. A previous study showed that the LncRNAs localized in the cytoplasm were involved in the modulation of mRNA stability [32], and these lncRNAs played a crucial role in fine-tuning and regulating gene expression via miRNAs and acting as competing endogenous RNAs (ceRNAs) [33]. *PSEN1* regulates the enzymatic cleavage of γ-secretase of APP protein and Notch [34,35]. In this study, we found that BP can regulate *PSEN1*. BP overlapped with γ-secretase inhibitor DAPT in its binding to the active site of the PSEN1 through molecular docking calculation. However, the PSEN1 binding affinity of BP was not as strong as that of γ-secretase inhibitor DAPT by molecular docking simulations (Figure 1D).

We found that the LncCYP3A43-2 levels were significantly reduced by BP treatment in Ts-21 neurons, and this resulted in a subsequent upregulation of miR-29b-2-5p, confirming the hypothesis that lncRNA acts as an miRNA sponge (Figure 1H,K). Likewise, our data demonstrated that the therapeutic potential of BP in treating AD was through regulating miRNA-29b-2-5p and consequently reducing *PSEN1* expression (Figure 2 and Figure 3). The β-CTF/C6 cells could overexpress *PSEN1* and Aβ that induce neurotoxicity and cause neuronal death. Therefore, this cell line is suitable for observing the action of drugs on *PSEN1* [11]. When miR-29b-2-5p was suppressed, BP no longer exerted its effect on PSEN1 (Figure 3G,H). These data provide strong evidence that BP acted via regulating lncRNA-miRNA-mRNA (lnc-CYP3A43-2 -miR-29b-2-5p-*PSEN1*) network and in turn reduced β-amyloid accumulation.

Herein, we are the first to present the therapeutic potential of BP in treating AD using a mouse model (3xTg-AD mice). Our results show that 3xTg AD mice treated with BP had significantly reduced Aβ accumulation in the hippocampus and cortex regions as compared to untreated controls, and this finding is further supported by our [18F]-FBB PET study (Figure 4D,F). The level of Aβ reduction in the cortex and hippocampus of 3xTg AD mice was related to the concentration of BP used in treatment. In specific, in the cortex, 60 mg/kg BP treatment could not inhibit Aβ accumulation, whereas 120 mg/kg BP treatment could. In the hippocampus, we observed a similar situation: 60 mg/kg BP treatment could maintain the Aβ level in the hippocampus, whereas 120 mg/kg BP treatment could decrease Aβ accumulation. Recent literature showed that in an animal model of Spinocerebellar Ataxia Type 3, BP induced autophagy and the elimination of aggregates [36]. Thus, we speculate that BP at high concentrations, such as 120 mg/Kg, can play a role in promoting autophagy for the elimination of Aβ in the hippocampus of 3xTg AD mice in addition to its primary function of reducing *PSEN1*. The 3xTg mice also produced p-Tau in the hippocampus and cortex regions detected by [18F]-THK5351 [37]. In this context, our data suggests that BP affects the levels of p-Tau in the cortex but not in the hippocampus, although this needs further studies to derive definite conclusions (Figure S9). The discrepancies in its effect on Aβ and p-Tau might imply that BP elicits its effects through distinct mechanisms in the cortex and hippocampus for treating AD. However, further investigations are needed.

We tested the effect of BP on the performance of 3xTg AD mice in the Morris water maze. In addition, we compared the efficacy of BP with the clinically used drug Donepezil. Donepezil is an FDA-approved cholinesterase inhibitor for treating AD. However, Donepezil’s mechanism does not directly involve PSEN1 and Aβ [38]. We found that the efficacy of BP is comparable to that of Donepezil when it comes to Aβ reduction. Our study suggested that BP facilitates spatial learning and memory in 3xTg AD mice (Figure 5).

We generated miR-29b-2-5p mutant mice using CRISPR/Cas9 technique. The results showed that the expression levels of *PSEN1* and Aβ were upregulated in the hippocampus of these mice (Figure 6A–E). This result supported the evidence regarding the role of miR-29b-2-5p in AD and its regulation by *PSEN1*. On the other hand, we showed that miR-29b-2-5p levels were downregulated in sample brain tissue of patients with AD (Figure 6F). This result suggested the critical role that miR-29b-2-5p plays in the pathogenesis of, and it deserves further investigation to see if cerebrospinal fluid samples from patients with AD have a similar trend in the future. In our study, miRNA-29b-2-5p was significantly decreased in the BA9 tissue samples of patients with Alzheimer’s.

In conclusion, we proposed that BP can effectively reduce amyloid production and promote cognitive function in the 3xTg AD mouse model. Furthermore, miR-29b-2-5p might be a good therapeutic target for treating early AD. This is attributed to its ability to regulate PSEN1 expression via lnc-CYP3A43-2/miRNA-29b-2-5p. However, some obstacles remain to be tackled when considering miRNA therapy for AD, including inappropriate biodistribution, disruption, and saturation of endogenous RNA machinery, and untoward side effects [39]. We propose modifying the structure of miR-29b-2-5p through wrapping it with lipophilic nanoparticles, exosomes, or combining tissue-specific targets in the hippocampus. These methods are expected to solve the problems of non-specificity to the brain and instability.

## 4. Methods and Materials

### 4.1. Culture of Ts21-iPSCs

The Ts21-iPSC cells were reprogrammed from allantoic fluid-derived trisomy 21 iPS cells as previously described [4]. These cells displayed the characteristics of AD phenotype with accumulated amyloid deposits and Tau protein hyperphosphorylation. The iPSCs were cultured in Essential 8 Medium (Thermo Fisher, A1517001, Waltham, MA, USA) containing 1% Essential 8 Flex Supplement (Thermo Fisher, A28584-01) and 1% penicillin-streptomycin (Thermo Fisher, 15140-122) [4]. When the cells reached complete confluence, they were washed once with 1× PBS before adding Accutase (Thermo Fisher, A1110501) to dissociate into small clumps and seeded on 1% Matrigel matrix (Corning, 354230, Corning city, NY, USA) for neural maturation as previously described [4]. The iPSCs were maintained at 37 °C in a 5% CO_2_ incubator, cell culture medium was replaced once daily. To promote the differentiation of Ts21 multifunctional stem cells into neuronal cells, they were treated with Accutase (Thermo Fisher, A1110501) and cultured in DMEM/F12 HEPES (Thermo Fisher, 11330032) medium containing KnockOut Serum Replacement (Thermo Fisher, 10828028). These cells displayed as suspension of embryoid bodies [4]. After one day, the medium was changed to high-glucose DMEM (Thermo Fisher, 11965092) and KnockOut DMEM/F-12 (Thermo Fisher, 12660012) containing nerve-inducing components and cultured for another two days. The medium was changed every two days until Day 30.

### 4.2. β-CTF/C6 Cells

The human C-terminal 99-residue fragment (β-CTF) of amyloid precursor protein was introduced into the C6 rat glioma cell line using a lentivector (System Biosciences LV500A-1, Palo Alto, USA) as previously described [11]. The production of Aβ40 and Aβ42 peptides was induced by a Cumate-inducible gene expression system (System Biosciences, PBQM100A-1) [11]. The culture medium was Ham’s F12K (Thermo Fisher, 21127022) containing 2.5% FBS, 15% horse serum (Thermo Fisher, 26050088), and 1% penicillin-streptomycin (Thermo Fisher, 15140122). The cell line was kindly provided by Professor Yi-Cheng Chen from the Department of Medicine, Mackay Medical College, Taipei, Taiwan.

### 4.3. SH-SY5Y Cell Model

The human SH-SY5Y cell line was isolated from SK-N-SH, a metastatic bone tumor biopsy cell line, and cultured for 5–7 days in DMEM (Thermo Fisher, 11965092) supplemented with 10% fetal bovine serum (FBS) (Thermo Fisher, 16000044). When the cells were grown to 80% confluence in the flask, the serum-containing medium was replaced with Neurobasal Medium (Thermo Fisher, 21103049), containing B27 supplement (Thermo Fisher, 17504044), GlutaMAX (Thermo Fisher, 35050061), and 10 µM retinoic acid (Merck, R2625, Darmstadt, Germany) to promote the differentiation and growth of neuronal phenotypes.

### 4.4. n-Butylidenephthalide

n-Butylidenephthalide (3-Butylidenephthalide, BP) was purchased from Sigma-Aldrich (purity ≥96%, #W333301, St. Louis, MO, USA) as a mixture of cis and trans isomers. The stock solution of BP was prepared in a mixture of 99% alcohol, chloroform, and pure water in the ratio of 1:1.5:1.5:6, respectively. Olive oil (Oro del, Desierto, Spain) was used to dilute the stock BP solution to the desired concentration for animal use. The reported effective concentrations of BP for neuronal degeneration in animal models were around 100~250 mg/kg, and did not shorten mice survival [40]. We chose the first effective concentration (120 mg/kg/day) to treat 3xTg AD mice, and the low concentration group was 60 mg/kg/day, they were administered orally. It was well documented that BP efficiently crossed the blood–brain barrier [41]. The vehicle group was treated with olive oil. Mice of the same age were randomly assigned to each group and subjected to the same training. These treatments on 3xTg-AD mice were started from the age of 4 to 14 months.

### 4.5. Cell Viability under BP Treatment

To examine the protective ability of BP against Aβ-induced toxicity, CTF/C6 cells were treated with a designed concentration of BP. For viability assay, 1 × 10^4^ CTF/C6 cells based on the result shown in Figure S6 in supplemental information, were incubated with 0, 50, 100, 150, and 200 μM of BP in a 96-well microtiter plate for 72 h at 37 °C incubator containing 5% CO_2_. The incubated cells treated with or without BP were then assayed for cell viability. Cell viability was assayed by adding 10 μL MTT solution to each well, and wells were incubated for another 2–4 h at room temperature. The optical density was determined at 450 nm by using a microplate reader (Thermo Fisher, Multiskan GO).

### 4.6. Molecular Docking Calculation

The binding energy or the molecular docking calculation between BP and PSEN1 protein was performed with the Lamarckian Genetic Algorithm (LGA) by Autodock 4.2 software [20]. The structures of Presenillin1 protein (PDB ID: 5FN2) [19] were obtained from the RCSB Protein Data Bank [42]. The co-crystallized ligands of protein were all removed. Besides, the protein structure was modified by adding polar hydrogens and Kallman united atom charges for docking calculation by AutoDock Tool 1.5.6 interfaces (ADT) [43]. BP was optimized with MMFF94 force field by ChemBio3D version 11.0 software (Cambridge Soft Corp, Cambridge, UK). Hydrogens and Gasteiger charges were also added to the ligand for the docking study by ADT. The Grid box calculated by AutoGrid program was centered at the x = 116.95, y = 123.86 and z = 125.29, with dimensions 52 × 76 × 58 Å grid points at a spacing of 0.375Å. All docking parameters were set to default except for the following parameter: maximum number of energy evaluation increased to 25,000,000 per run. The docking results were analyzed with cluster analysis, and the simulation model was shown by BIOVIA Discovery Studio 4.5 Visualizer software (BIOVIA Corp., San Diego, CA, USA). AutoDock analysis was conducted by Dr. Yang-Chang Wu.

### 4.7. Predicting miRNA Interaction That Could Interfere with Gene Expression

miRNA interaction at the 3′-end of the gene which interfere with the gene expression (Target detection) was performed using the software RNA22 v2 miRNA.

### 4.8. Predicting lncRNA Interaction That Could Interfere with miRNA Expression

Target detection was performed using the software of Freiburg RNA Tools to predict the lncRNA interaction that could interfere with miRNA expression.

### 4.9. Microarray

The cells were treated with Trizol reagent (Thermo Fisher, 15596-018), and the miRNA of Ts21 neurons was detected using the human miRNA microarray chip (Agilent Technologies, G4872A-070156, Santa Clara, CA, USA). Each group was subjected to three replicates, and miRNAs exceeding two-fold expression levels were considered significant. Long noncoding RNA microarray were purchased from Agilent (Agilent Technologies, G4851C). Microarray analysis was conducted to evaluate expression levels of these lnRNA between the BP-treated and untreated (control) groups.

### 4.10. Extracellular Protein Measurement by Sandwich ELISA

After neuronal differentiation of Ts21 iPSCs (5 × 10^5^ cells), the conditioned mediums were collected and centrifuged at 1200 rpm for 5 min. These supernatants were stored at −80 °C. They were analyzed by sandwich ELISA (IBL-international, 27711, Hamburg, Germany), and Aβ42 quantitation were carried out within 30 min (Thermo Scientific, Multiskan GO).

### 4.11. Real-Time PCR

Primers were prepared and diluted to 20 μM/μL, and cDNA samples were diluted to 50 ng/μL. A 96-well plate was ready for RT-PCR. and then, 10 μL SYBR Green PCR Master Mix (Thermo Fisher, A25741), 2 μL cDNA, 2 μL primer, and 6 μL RNase-free water were added to each well and gently mixed. Gene expression was detected using the QuantStudio™ 3 Real-Time PCR System. The primers for mRNA are listed in Table 3.

### 4.12. Western Blot Analysis

Cells were analyzed by western blotting using 4–12% SDS gradient gels (NP0322PK2; Invitrogen) and the following antibodies were used: APP (Abcam, ab32136, Cambridge, UK), BACE1 (Abcam, ab183612), PSEN1 (Cell Signaling Technology, D39D1, Danvers, MA, USA), cleaved Notch1 (Cell Signaling Technology, 4147), Aβ1-42 (Abcam, ab201060), and anti-β-actin (Abcam, ab8227). Electrophoreses were performed using 15% Tris-Glycine SDS gels for Aβ and 12% Tris-Glycine SDS gradient gels for PSEN1 in 12-well gel, and the resulting bands were transferred by western blotting. The protein concentration of the loading sample was 30 μg. Immunoreactivity was analyzed by chemiluminescence (GE Healthcare, Piscataway, NJ, USA). The protein marker ranges from 10–180 kDa were used (Tris-glycine-SDS running buffer) (GeneDireX, PM006-0500, Taoyuan City, Taiwan). The transfer buffer was 25% Methanol and 10% TG-SDS buffer (Avantor, 0783-5L, Radnor, PA, USA). The setup time and voltage are 90 min and 400 mA by gel transfer to PVDF member. The chemiluminescence signal was observed using a digital image analyzer (Fuji Film Inc, LAS-3000, Tokyo, Japan).

### 4.13. Transfection

One hundred microliter transfection solutions were prepared and supplemented with 10 nM miRNA-29b-2-5p mimic or miRNA-29b-2-5p -siRNA in a 4.5:1 mixture. They were then incubated for 10 min at 23 °C before cell transfection (Lonza, V4XP-3024, Basel, Switzerland). Cells were trypsinized and counted, 1.0 × 10^6^ cells were then seeded in six-well culture plate. After centrifugation at 335× *g* for 5 min, the respective siRNA solution was mixed with cells and placed in a tube shocker. The cells were transfected by electroporation (Lonza, V4XP-3024) and transferred to a new six-well culture plate to be cultured overnight in an incubator at 37 °C with 5% CO_2_. Before transfection, 75 pmol of biotinylated hsa-29b-2-5p miRNA was diluted in 200 μL complete neuron basal (NB) medium (Thermo Fisher, 21103049). This mixture was transferred to another 1.7 mL microfuge tube containing Lipofectamine 3000 reagent (Thermo Fisher, L3000001) diluted in 200 μL of Opti-MEM media (Thermo Fisher, 31985088). These cells were again cultured at 37 °C with 5% CO_2_ for at least 36 h. The preparation of lnc-CYP3A43-2 siRNA and miR-29b-2-5p-i experiments were the same as stated above. The miR-29b-2-5p-i sequence: 5′-CUAAGCCACCAUGUGAAACCAG-3′.

### 4.14. Flow Cytometry

β-CTF/C6 cells emit endogenous fluorescence after activation by Cumate (System Biosciences, QM100A-1). For analysis, 5 × 10^5^ cells were washed in 500 μL PBS and centrifuged at 1500 rpm for 5 min. The supernatant was discarded, and the centrifugation was repeated once more. The centrifuged cells were resuspended in 500 μL PBS and evaluated using a flow cytometer (Beckman, Coulter CytoFLEX S Flow Cytometry, Brea, CA, USA).

### 4.15. Dual-Luciferase Assay System

Primers were annealed and digested with Sacl and Xbal restriction enzymes. The digested fragment was purified and cloned into pmirGLO Dual-Luciferase miRNA Target Expression Vector (Promega, E1330, Madison, WI, USA). The cloned fragment included the miR-29b-2-5p targeted PSEN1 sites (Figure 2). The reporter gene was transfected using the following Dual-Luciferase Reporter Assay System (Promega, E1910): (1) firefly luciferase (*Photinus pyralis*) with a molecular weight of 61 kDa and OD measurement at approximately 560 nm and (2) *Renilla* luciferase (*Renilla reniformis*) with a molecular weight of 31 kDa and OD measurement at approximately 480 nm. After transfection into cells, fluorescence was emitted without stimulation by drugs. The comparison of luminescence intensity revealed the expression of the reporter gene.

### 4.16. Biotin-Based Pulldown Assay

For this assay, 1 × 10^6^ Ts21-neuron cells were seeded and cultured overnight at 37 °C in a 5% CO_2_ incubator. Thereafter, a solution containing 75 picomoles of biotinylated miRNA was prepared in a total 200 µL MEM. This mixture was transferred to another microfuge tube containing Liposome-based transfection reagent (8 µL/well) diluted in 200 µL MEM. They were mixed gently and incubated at room temperature to allow for the formation of the transfection complexes. After that, the transfection complexes were added in a drop-wise manner to the cells, and they were cultured for at least 36 h. Streptavidin Coated Magnetic Beads (Merck Millipore, LSKMAGT10, Burlington, MA, USA) were prepared, and 100 µL of bead wash buffer (10 mM Tris-Cl pH 7.5, 0.5 mM EDTA, 1 M NaCl) was added to the beads and washed by vortexing at room temperature. The tube containing beads was then placed on the magnet for 2 min. The resulting supernatant was carefully removed and discarded, and the previous step was repeated three times. Next, 100 µL of RNase freeing solution (0.1 M NaOH, 0.05 M NaCl) was added to the beads and mixed, and the supernatant was discarded. This step was repeated twice more. Bead resuspension solution (0.5 M NaCl) was added, and the mixture was vortexed and incubated at room temperature for 5 min. The resuspended beads were placed on the magnet for 2 min, and the supernatant was removed. After that, 200 µL of bead blocking solution (1 µg/µL BSA) was added, and they were incubated at 4 °C for over-night on a multi-tube rotator. Transfected Ts21-neuron cells were harvested by gentle scraping using a sterile cell scraper within a laminar hood, each sample was placed into a new microfuge tube. Complete pull-down wash buffer (10 mM KCl, 1.5 mM MgCl2, 10 mM Tris-Cl pH 7.5, 5 mM DTT, 1 M NaCl, 0.5% IGEPAL, 60 U/mL Superase, and 1× Protease Inhibitor cocktail) were prepared. 150 µL of ice-cold CPDW buffer was added to the beads, vortexed, and incubated at room temperature for 30–60 s. The supernatant was removed and discarded, and this step was repeated two more times. The beads were resuspended in 100 µL nuclease-free water and incubated on ice. Total RNAs were extracted, cDNA synthesis was performed and subjected to qPCR as previously described [44].

### 4.17. AD Animal Model

Experiments were conducted using transgenic mice (3xTg) harboring the following three transgenes: presenilin PS1M146V knock-in mutation (PS1^M146V^), human transgenes and the APP Swedish mutation (APPSwe), and microtubule-associated protein tau (tauP30IL) (The Jackson Laboratory, Sacramento, CA, USA). As these mice age, they gradually exhibit synaptic dysfunction neuropathy; Aβ protein accumulates in the later stage, and plaques and neurofibrillary tangles are formed. At the age of 3–6 months, Aβ deposits are detected in some regions of the brain. At the age of 12 months, the pathological features include highly phosphorylated tau protein and neurofibrillary tangles in the hippocampus. In this study, only female mice were used.

### 4.18. Gene-Edited for miR29b-2-5p in C57BL/6 Mouse

Mouse zygotes were obtained by mating super-ovulated B6 females and males (National Laboratory Animal Center, Taiwan). Thus, sgRNA, Cas9 nuclease, and ssDNA were mixed just before the microinjection into the pronuclei of zygotes. The injected embryos were incubated at 37 °C until they were transferred into the pseudopregnant females on the same day. Mouse genomic DNA was extracted from the tail tips of pups, and the genomic sequences around the mutant site were PCR-amplified using the following primers: miR29b-2-5p F 5′-ACAGTTTCTTGTGCAGACATCGG-3′, Chchd23B 5′-GGGACCACTTCTCATTGCCATAG-3′. The obtained PCR products were directly sequenced. The animals were kindly provided by Professor Si-Tse Jiang of the National Laboratory Animal Center, Taiwan. miR-29b-2-5p-mutant mice were obtained from the National Laboratory Animal Center, National Applied Research Laboratories, Taiwan (109-NLAC-AA-017).

### 4.19. PET-CT Imaging

The accumulation of Aβ deposits in 3xTg mice occurs over a long period of time. Using [18F]-Florbetaben (FBB) or [18F]-THK5351 PET scan, the accumulated amyloid-like protein in the brain can be observed without sacrificing the animals. Mice were anesthetized by inhalation of 2–3% isoflurane and then injected with [18F]-FBB (3 mCi) through tail vein. We waited for 30 min to allow for [18F]-Florbetaben or [18F]-THK5351 uptake, and then static PET images were recorded for a total of 30 min using Sedecal SuperArgus PET 2r (Sedecal, Madrid, Spain). List mode data were acquired for 30 min per scan using an energy window of 250–700 keV. To improve resolution and sensitivity, the acquired images were reconstructed using the 2D OSEM algorithm. The image voxel size was 0.387 × 0.397 × 0.775 mm. Quantitative image analysis of the mouse brain was performed using PMOD (PMOD Technologies, v3.8, Zürich, Switzerland). Mouse brain images were aligned to a standard mouse brain template using PMOD’s PFUS module rigid matching tool. The regions of interest of each mouse brain, including the frontal cortex, hippocampus, and cerebellum, were then manually defined and expressed as standard uptake values. The SUVR of each brain region was expressed as cortex/cerebellum and hippocampus/cerebellum. All SUVRs were calculated using the cerebellum as a reference region.

### 4.20. Immunohistochemistry Staining

Brain slices obtained from four mice in each group were simultaneously stained with purified anti-β-Amyloid antibody (Cell Signaling, #9888) by immunohistochemistry. Briefly, after 20 min of antigen retrieval using 88% formic acid at room temperature, the slices were mixed with 500× diluted Aβ antibody and incubated overnight at 4 °C. This was followed by hematoxylin (Leica, 3801522, Wetzlar, Germany) counterstaining, and DAB (TA-060-QHSX and TA-002-QHCX, Thermo Fisher) was used as a chromogen for detection. The photographs of Aβ staining were captured using a microscope (Olympus, VS120-S6, Tokyo, Japan). Amyloid-beta 1-42 plaque load was analyzed only in the CA1 to CA3 of the hippocampus region. The quantification of Aβ deposits was performed using the ImageJ software (National Institutes of Health, Bethesda, ML, USA). Thioflavin S (Th-S) staining was performed in which Th-S (Sigma-Aldrich, #230456) was prepared with distilled water. The tissue slides were first deparaffinized and hydrated with distilled water and then stained with hematoxylin for 5 min. They were washed with running water for 5 s and rinsed with distilled water. Finally, they were soaked in 1% Th-S for 5 s at room temperature, differentiated in 70% alcohol for 5 s, and rinsed in distilled water. In addition, 6E10 staining was also performed, during which slides were incubated with 3% H_2_O_2_ solution for 10 min to quench endogenous peroxidase activity at room temperature. They were rinsed later with 1× TBS and blocked using a blocking buffer. Finally, the slides were incubated with 6E10 (Biolegend, SIG-39320, San Diego, CA, USA) at room temperature, rinsed with 1× TBS, and labeled with the secondary antibody.

### 4.21. Quantification of Hippocampus miRNA Expressions in 3xTg and miR-29b-2-5p Mutant Mice

The analysis of hippocampus miRNAs was conducted on 6-month-old female miR-29b-2-5p mutant mice (*n* = 4 for each group) and 14-month-old female 3xTg mice (*n* = 7 for each group). All animal procedures have been approved by the animal committee of Hualien Tzu Chi Hospital and Tzu Chi University (#108-04, Hualien Tzu Chi hospital). Total RNA was extracted from hippocampus using RNeasy Mini kit (Qiagen, 74106, Hilden, Germany), and miRNAs were purified using the miRNeasy Mini Kit (Qiagen, 217004). cDNAs were synthesized using the TaqMan Advanced miRNA cDNA Synthesis Kit (Thermo Fisher, A28007). The primer pairs purchased were the mmu-miR-29b-2-5p (Thermo Fisher, mmu481675_mir) and mmu-miR-124-3p (Thermo Fisher, mmu480901_mir,). The quantification of these miRNAs was conducted by real-time PCR (Thermo Fisher, A28567).

### 4.22. Morris Water Maze Evaluation of Learning and Memory

Mice were trained in a swimming pool with a rest platform hidden under opaque water for 5 days. On Day 1, 120 s were allowed for mice to find the platform, and if the mice could not find the platform on time, we led them to the platform. On Days 2–5, the time allowed to reach the rest platform was decreased to 90 s; this training was also conducted twice. On Day 5, the time required to reach the rest of the platform was recorded. The water maze analysis system used in this study was Smart V3.0 Basic Pack.

### 4.23. Quantification of miRNA Expression in Human or Mouse Brain

Frozen brain specimens isolated from the BA9 of the frontal cortex were obtained from the NIH NeuroBioBanK. Control participants (*n* = 6) and patients with AD (*n* = 6) were referred by Dr. Catherine Cahill (NBTR NeuroBioBank Req, #1311, Bronx, NY, USA). Specimens were stored at −80 °C. Human brain BA9 tissue specimens were examined at Harvard Medical School, Massachusetts General Hospital. The information on the human brain samples of controls and patients with AD are listed in Table 4. RNA was purified using the miRNeasy MiniKit (Qiagen, 217004), and cDNA was synthesized using the TaqMan Advanced miRNA cDNA Synthesis Kit (Thermo Fisher, A28007). The product was detected using real-time PCR and quantified. The targets included hsa-miR-29b-3p (Thermo Fisher, 478369_mir), hsa-miR-29b-2-5p (Thermo Fisher, 478003_mir), hsa-miR-9-5p (Thermo Fisher, 000583_mir). The expression was quantified in absolute terms as miRNA copy counts/5 ng of total RNA, and hsa-miR-9-5p was used as an internal control. Regular cDNA synthesis from mRNA was performed using either RevertAid RT Kit or SYBR Green Master Mix (Thermo Fisher, 4368577).

### 4.24. Statistical Analysis

ANOVA was performed for the comparison between multiple groups. LSD methods were used as post hoc tests for pairwise comparisons, and Dunnett’s tests compared a fixed group and other groups. Repeated measures ANOVA was performed for the comparison of subjects with multiple measurements. The student’s *t*-test was performed to compare two groups. All tests were two-tailed, and data sets were statistically significant when *p* < 0.05. Statistical analyses were performed using IBM SPSS 20.0.0 software.

## Figures and Tables

**Table 1:** Prediction of lncRNA-microRNA interaction.

lncRNA/miRNA	lnc-P2RY2-3:1			lnc-CYP3A43-2:1			lnc-ZBTB7C-1:1		
	lncRNA Start-End	miRNA Start-End	Energy	lncRNA Start-End	miRNA Start-End	Energy	lncRNA Start-End	miRNA Start-End	Energy
miR-29b-2-5p	113–119	10–16	−2.69	204–219	3–19	−11.49	11–22	9–20	−7.86
miR-26b-5p	68–75	13–20	−8.66	230–240	10–20	−7.07	433–439	4–10	−1.85
miR-99a-5p	5–12	6–13	−7.67	386–392	15–21	−4.64	319–-325	16–22	−2.86

**Table 2:** Prediction of miRNA-29b-2-5p—*PSEN1* interaction.

miRNA Identifier	Energy (−kcal/mol)	Predicted Target Site of *PSEN1*	Base Pairs in Outative Heteroduplex	Span of Target (A=T, G≡C)	*p*-Value
has-miR-29b-2-5p	−12.5	TCATAGTGCGTTGTGAAATGGC	16	20	0.0775
	−14.7	AGACATCTGCATGTGATCATCT	17	21	0.0171

A lower *p*-value represents a greater chance that the loci contain a valid miRNA recognizing element. Sequence and predicted base pairing of human miR-29b-2-5p with *PSEN1* (red color).

**Table 3:** Primer Sequences.

Gene	Primer	Sequence (5′ to 3′)
*APP*	Forward	5′-ATGAGCTGCTTCAGAAAGAGC-3′
	Reverse	5′-TTCGTTTTCTGTGTTGGCTGGC-3′
*BACE1*	Forward	5′-CTTTGACTCTCTGGTAAAGC-3′
	Reverse	5′-TCACCTCATAATACCACTCC-3′
*β-actin*	Forward	5′-CACCTTCTACAATGAGCTGC-3′
	Reverse	5′-ATCACAATGCCTGTGGTACG-3′
*GAPDH*	Forward	5′-AACATCAAATGGGGTGAGGC-3′
	Reverse	5′-TTGTCATGGATGACCTTGGC-3′
*lncCYP3A43-2*	Forward	5′-ATCGAAGCAGGCAGCCTTGC-3′
	Reverse	5′-TGGAGCTCCCTCTGAAGGAC-3′
*PSEN1(Human)*	Forward	5′-AAATCACAGCCAAGATGAGC-3′
	Reverse	5′-GTGGACTACATTACTGTTGC-3′
*PSEN1(Mouse)*	Forward	5′-TAAGACCTAC AATGTCGCCG-3′
	Reverse	5′-AATCACAGCCAAGATGAGCC-3′
*PSEN2*	Forward	5′-TGTCACTCTGTGCATGATCG-3′
	Reverse	5′-TGGTCATAACCACGATGACG-3′

**Table 4:** The information on the human brain of control and AD.

**Group**	**Control**					
**Sex and Race**	**Female and White**					
No.	1	2	3	4	5	6
Age	84	83	89	85	80	85
Post mortem interval (minute)	1110	370	140	300	285	316
**Group**	**AD**					
**Sex and Race**	**Female and White**					
No.	7	8	9	10	11	12
Age	82	90	88	83	82	87
Post mortem interval (minute)	375	200	210	255	340	870

## Data Availability

The data that support the findings of this study are available from the corresponding author upon reasonable request.

## Supplementary Materials

The following supporting information can be downloaded at: https://www.mdpi.com/article/10.3390/ijms231810554/s1.
