# Supplementary material for: Targeting PSEN1 by lnc-CYP3A43-2/miR-29b-2-5p to Reduce β Amyloid Plaque Formation and Improve Cognition Function
Source: Int J Mol Sci. 2022 Sep 11;23(18):10554. doi: 10.3390/ijms231810554 (PMC9506169; doi:10.3390/ijms231810554)
Supplement: Supplementary file 1 [file ijms-23-10554-s001.zip › ijms-1865057-supplementary.pdf]

Article

Targeting *PSEN1* by lnc-CYP3A43-2/miR-29b-2-5p to Reduce β Amyloid Plaque Formation and Improve Cognition Function

Wei Wuli [1,2], Shinn-Zong Lin [1,3], Shee-Ping Chen [4], Bakhos A. Tannous [5], Wen-Sheng Huang [6], Peng Yeong Woon [7], Yang-Chang Wu [8,9,10], Hsueh-Hui Yang [11], Yi-Cheng Chen [12], Renata Lopes Fleming [5], Jack T. Rogers [5], Catherine M. Cahill [5], Tsung-Jung Ho [13,14,15], Tzyy-Wen Chiou [2,*] and Horng-Jyh Harn [1,16,*]

Supplementary Figure S1.

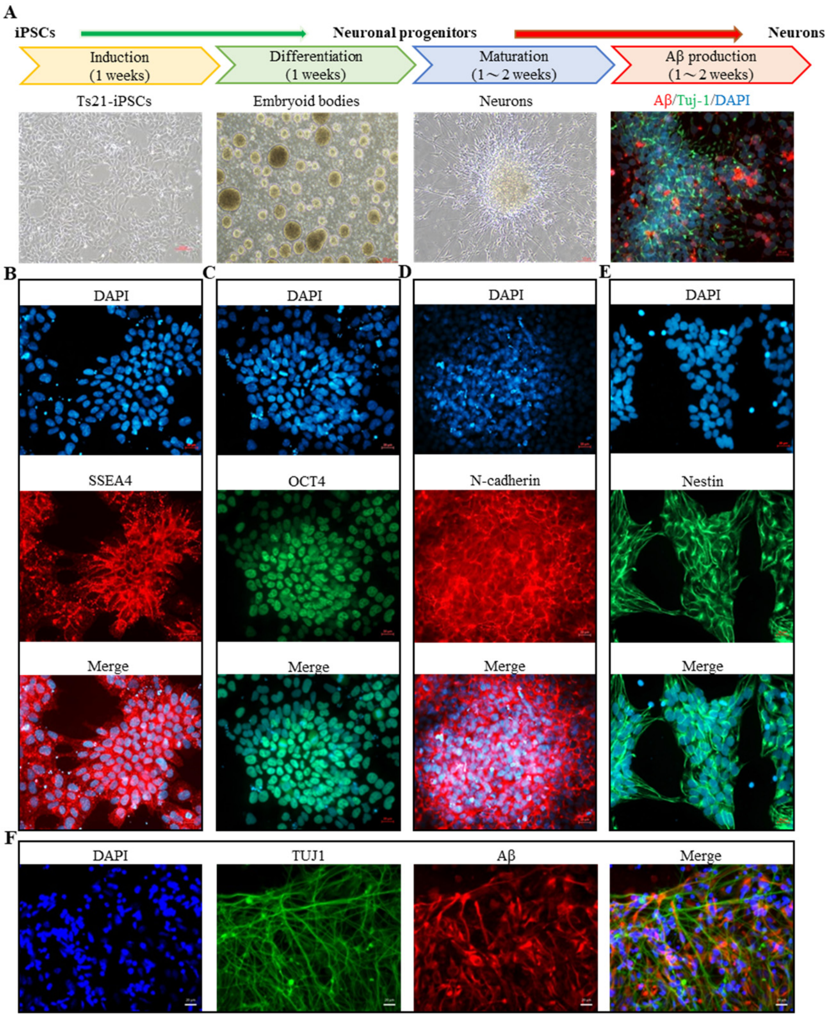

Supplementary Figure S1. (**A**) Schematic representation of the progression of Ts21-iPSC differentiation neurons. Ts21-iPSC colonies can be cut into fragments to form embryoid bodies. The embryoid bodies differentiated into mature neurons in the presence of a neuronal differentiation medium within 3–4 weeks. High-level neuronal expression of Aβ1–42 of Ts21-iPSCs in 5–6 weeks. (**B**) Ts21-iPSCs expressed stem cell marker, including stage-specific embryonic antigen-4 (SSEA4) and (**C**) octamer-binding transcription factor 4 (OCT4). The cell expressed neuronal progenitor cell-specific markers, namely, N-cadherin (**D**) and nestin (**E**) on differentiation to neuronal cells on Day 15. (**F**) Aβ production by human Ts21-iPSC-derived neurons. Blue, DAPI; green, TUJ1 (neuron-specific class III beta-tubulin); red, Aβ. All scale bars represent 20 μm.

Supplementary Figure S2.

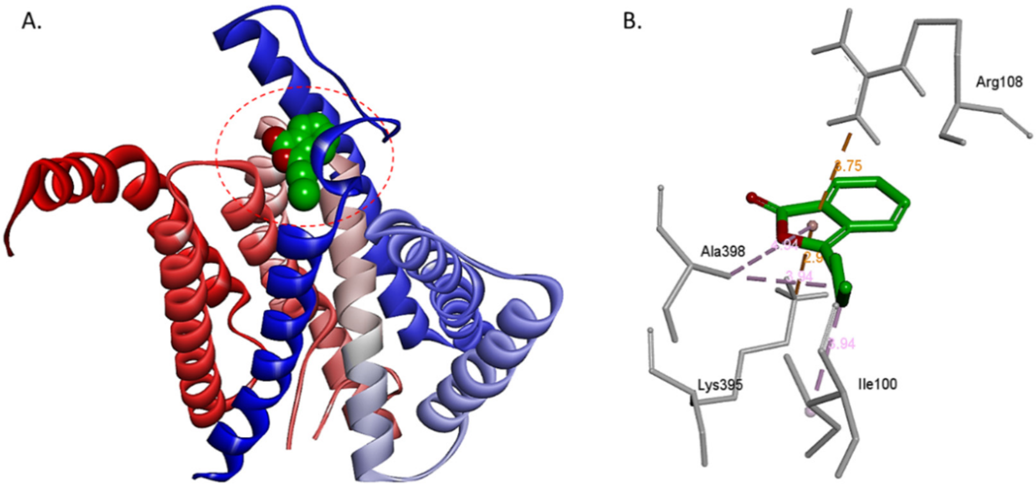

Supplementary Figure S**2.** (**A**) The simulation model showed that BP (green CPK) docked into the active site (dashed red ring) of Presenillin1 (ribbon). (**B**) In the active site, BP formed two pi-cation interactions (dashed orange line), a pi-alkyl interaction (dashed purple line), and two alkyl interactions (dashed light purple line) with amino acids of the Presenillin1 active site.

Supplementary Figure S3.

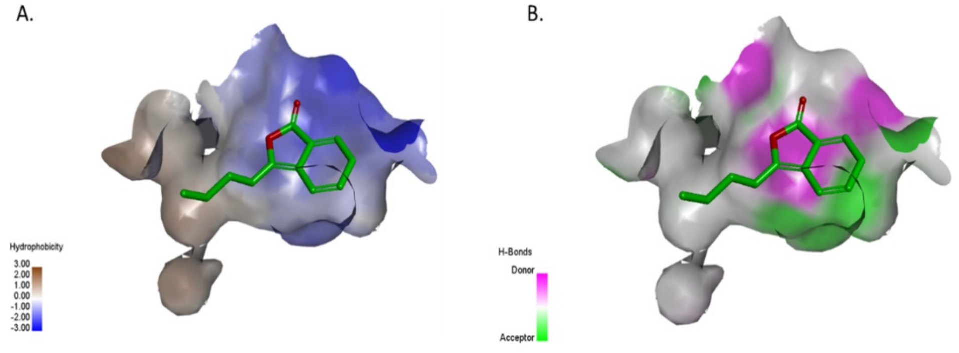

Supplementary Figure S**3.** The property distribution of amino acids around the docked BP (green stick). (**A**) The distribution of hydrophobicity amino acids was shown on the surface of the protein. The color browner meant the more hydrophobicity on the surface of amino acid residues. (**B**) The distribution of forming hydrogen-bonding properties on the surface of the protein. The color more pinking meant the properties of hydrogen bonding donor stronger on the amino acids.

Supplementary Figure S4.

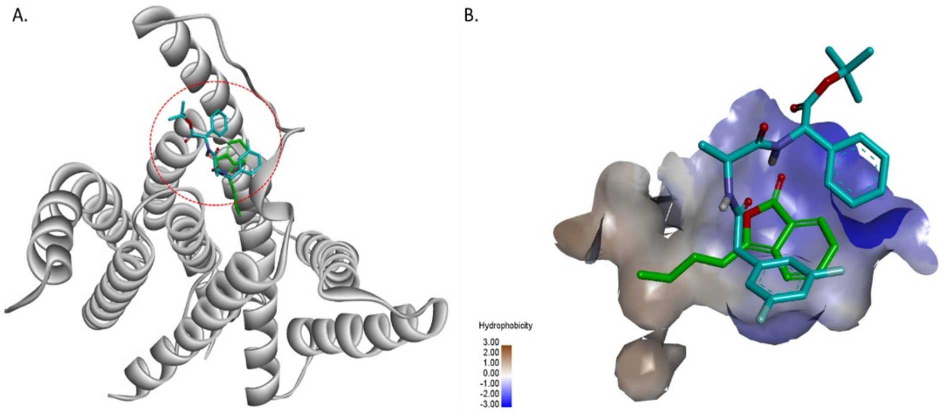

Supplementary Figure S4. The structure overlapping simulation between BP (green stick) and DAPT (cyan stick). (**A**) BP overlapped with DAPT in the active site of the Presenillin1 (gray ribbon). (**B**) BP overlapped with DAPT in the active site of the Presenillin1 (surface model). The color more pinking meant the properties of hydrogen bonding donor stronger on the amino acids.

Supplementary Figure S5.

A.

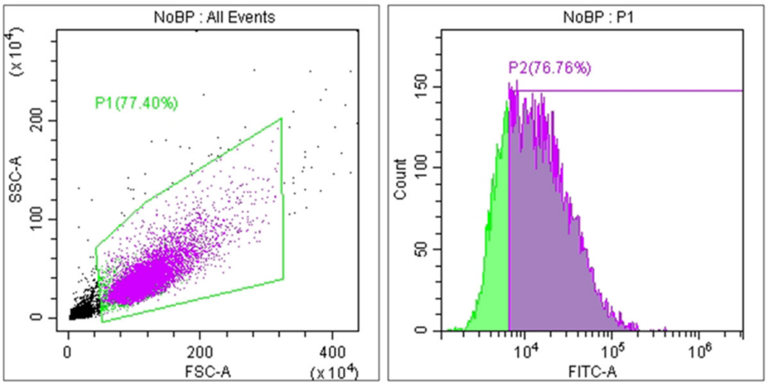

B.

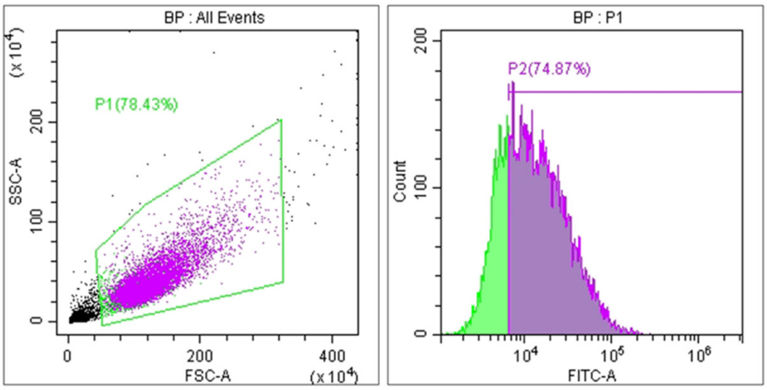

Supplementary Figure S5. β-CTF/C6 cells produced green fluorescent through Cumate production and detection the percentage by Flow cytometry. (**A**) Only Cumate induced the β-CTF/C6 cells to produce green fluorescent. The fluorescent expression 76.78% on cells. (**B**) The β-CTF/C6 cells treated with 600 μg Cumate and 100 μM of BP for 72 h. The fluorescent expression 74.87% on cells.

Supplementary Figure S6.

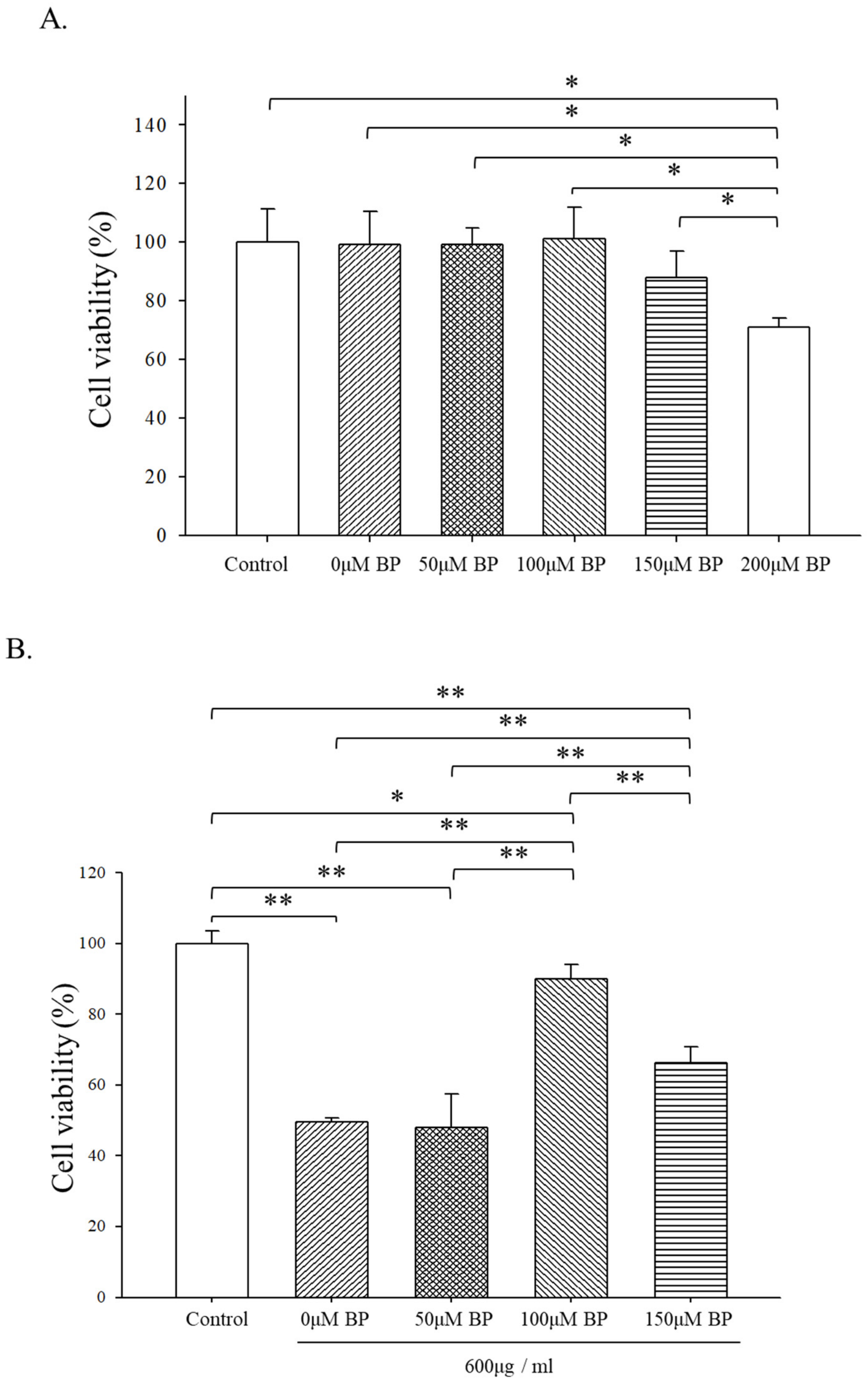

Supplementary Figure S6. Effect of BP on cell viability of β -CTF/C6 cells by MTT assay. (**A**). The effect of survival rate of β-CTF/C6 cells with different concentrations of BP. Control = 100 % ± 11.32, BP dilution solvent (0μM BP) = 99.22 % ± 11.16, 50μM BP = 99.22 % ± 5.7, 100μM BP = 101.02 % ± 10.7, 150μM BP = 87.89 % ± 9.01, 2000μM BP = 71.1 % ± 2.83. (**B**). Pretreated BP before Cumate induced β-CTF/C6 cells. Control = 100% ± 3.55%, Treated with 600 μg/ml Cumate (0μM BP) = 49.48 % ± 1.21%, 50 μM BP + 600 μg/ml Cumate = 48.04% ± 9.45%, 100 μM BP + 600 μg/ml Cumate = 89.95% ± 4.05%, 150 μM BP + 600 μg/ml Cumate = 66.32% ± 4.47%. *, $p < 0.05$. **, $p < 0.01$.

Supplementary Figure S7.

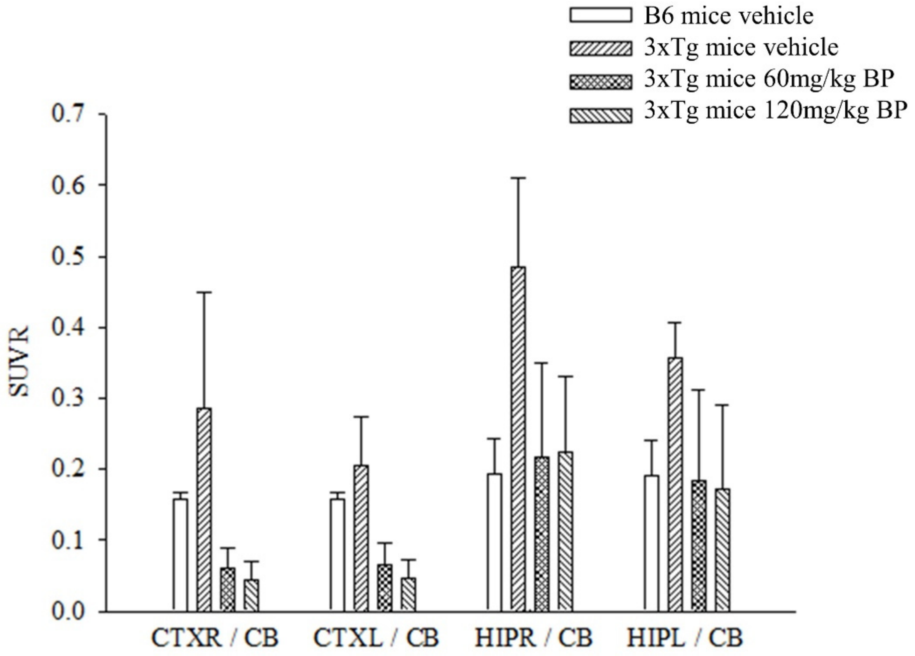

Supplementary Figure S7. The Aβ accumulation in the left and right sides of 3xTg mice brains (aged 4 months). VOI-based 18F-Florbetaben SUVR (cortex/cerebellum & hippocampus/cerebellum) comparison between B6 mice vehicle, vehicle-treated 3× Tg transgenic mice, and BP mice (60 or 120 mg/kg) aged 4 months (mean ± SD). SUVR, standard uptake value ratio; cortex, CTX; hippocampus, HIP; L, left; R, right. n = 2 for B6 mice, vehicle-treated 3xTg transgenic mice. n=3 for BP mice (60 or 120 mg/kg/day).

Supplementary Figure S8.

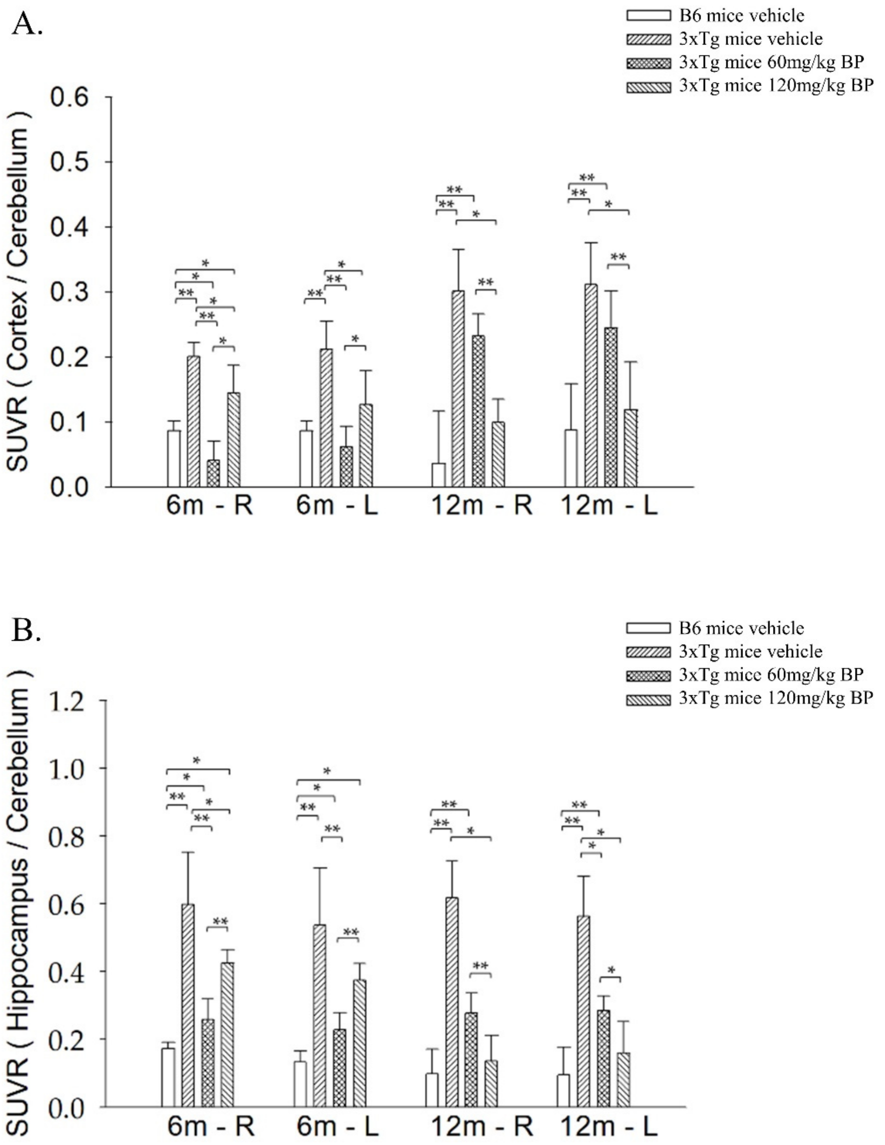

Supplementary Figure S8. The Aβ accumulation in the left and right sides of 3xTg mice brains (aged 6 & 12 months). (**A**,**B**) VOI-based ^{18}F-Florbetaben SUVR (cortex/cerebellum & hippocampus/cerebellum) comparison between B6 mice, vehicle-treated 3× Tg transgenic mice, and BP mice (60 or 120 mg/kg) aged 6 and 12 months. 6m-SUVR group: B6-CTX/CB-R& L = 0.08 ± 0.01 & 0.08 ± 0.01, B6-HIP/CB-R& L = 0.17 ± 0.01 & 0.13 ± 0.03, n=4. 3xTg mice vehicle -CTX/CB-R& L = 0.20 ± 0.02 & 0.21 ± 0.04, 3xTg mice vehicle-HIP/CB-R& L = 0.59 ± 0.15 & 0.53 ± 0.16, n=5. 60mg/kg- CTX/CB-R& L = 0.40 ± 0.02 & 0.61± 0.03, 60mg/kg-HIP/CB-R& L = 0.25 ± 0.06 & 0.22 ± 0.05, n=5. 120mg/kg- CTX/CB-R& L = 0.14 ± 0.04 & 0.12± 0.05, 120mg/kg-HIP/CB-R& L = 0.42 ± 0.03 & 0.37 ± 0.04, n=5. 12m-SUVR group: B6 mice-CTX/CB-R& L = 0.03 ± 0.07 & 0.08 ± 0.07, WT-HIP/CB-R& L = 0.09 ± 0.07 & 0.09 ± 0.08, n=4. Vehicle-CTX/CB-R& L = 0.30 ± 0.06 & 0.31 ± 0.06, Vehicle-HIP/CB-R& L = 0.61 ± 0.10 & 0.56 ± 0.11, n=5. 60mg/kg- CTX/CB-R& L = 0.23 ± 0.03 & 0.24± 0.05, 60mg/kg-HIP/CB-R& L = 0.27 ± 0.05 & 0.28 ± 0.04, n=5. 120mg/kg- CTX/CB-R& L = 0.09 ± 0.03 & 0.11± 0.07, 120mg/kg-HIP/CB-R& L = 0.13 ± 0.07 & 0.16 ± 0.09, n = 5.

Supplementary Figure S9.

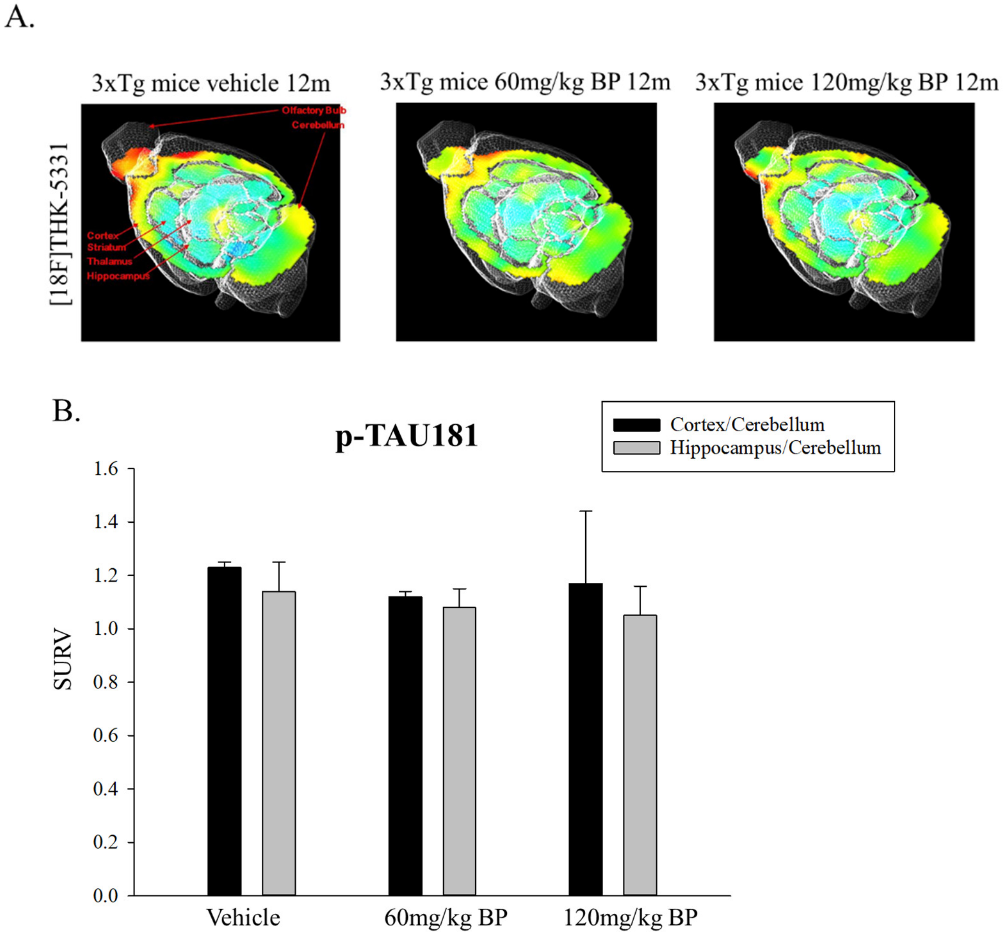

Supplementary Figure S9. The Tau-181 in the Cortex and Hippocampus of 3xTg mice brains (aged 12 months). (**A**) 3D radiotracer [18F]-THK5351 images show p-TAU181 in the brains of 3xTg mice after 12 months of birth. The 3xTg transgenic mice demonstrated p-TAU181 increased in the cortex (CTX) of the brain (orange-red color). Oral administration of BP (60 or 120 mg/kg) resulted in low levels of THK-5351(yellow-green color). But the images of the hippocampus didn't show a significantly different in the brain of 3xTg mice after 9 months of birth. (**B**) VOI-based ^{18}F-THK5351 SUVR (cortex/cerebellum & hippocampus/cerebellum) comparison between vehicle-treated 3× Tg transgenic mice and BP-treated 3× Tg mice (60 or 120 mg/kg) aged 9 months. 3xTg mice vehicle -CTX/CB = 1.23 ± 0.02, 60mg/kg- CTX/CB = 1.12 ± 0.02, 120mg/kg- CTX/CB = 1.17 ± 0.27. 3xTg mice vehicle-HIP/CB = 1.14 ± 0.11, 60mg/kg-HIP/CB = 1.08 ± 0.07, 120mg/kg-HIP/CB = 1.05 ± 0.11. n = 3 in each group. SUVR; standard uptake value ratio, M: months, Cortex: CTX, Hippocampus: HIP, cerebellum: CB.

Supplementary Figure S10.

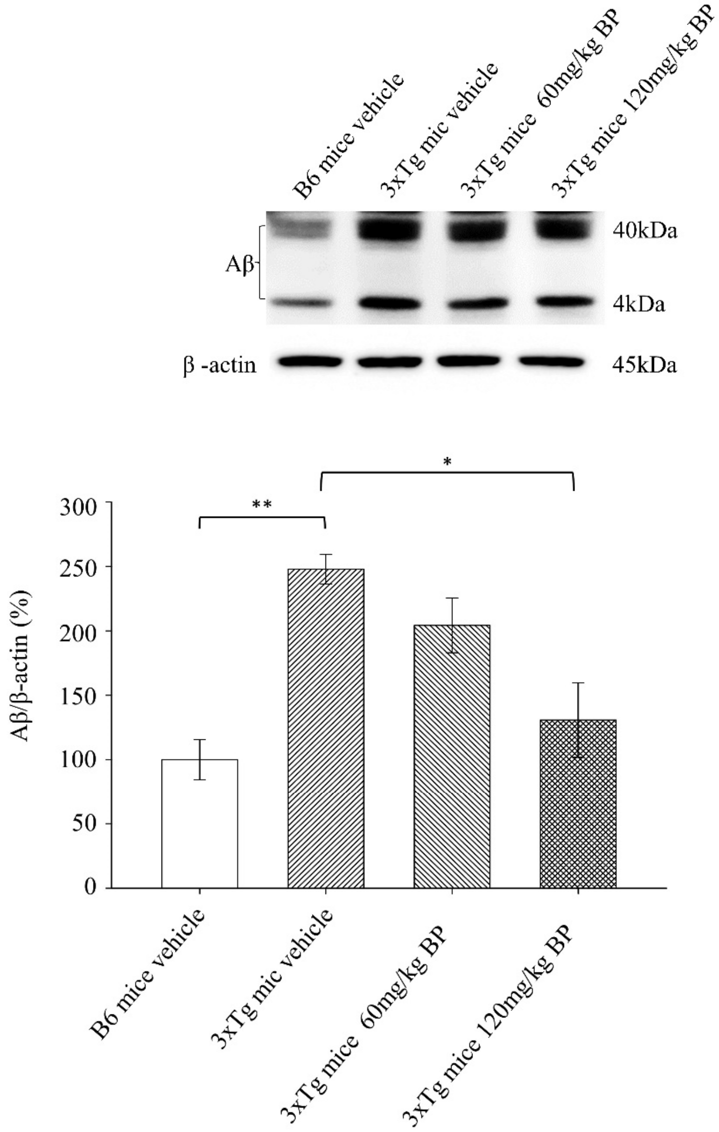

Supplementary Figure S10. Western blot analysis of Aβ in the hippocampus of BP treated 3xTg AD mice. The Aβ expression comparison between B6 mice, Oil treated 3xTg AD mice (3xTg mice vehicle), and oral BP mice (60 or 120 mg/kg) aged 14 months. The analysis result by Western blot, B6 mice = 100% ± 15.81 vs. 3xTg mice vehicle = 247.9% ± 11.51 vs. 3xTg mice 60 mg/kg = 204.28% ± 21.12 vs. 3xTg mice 120 mg/kg = 130% ± 28.47, n = 4 in each group. *$p < 0.05$. **$p < 0.01$.

Supplementary Figure S11.

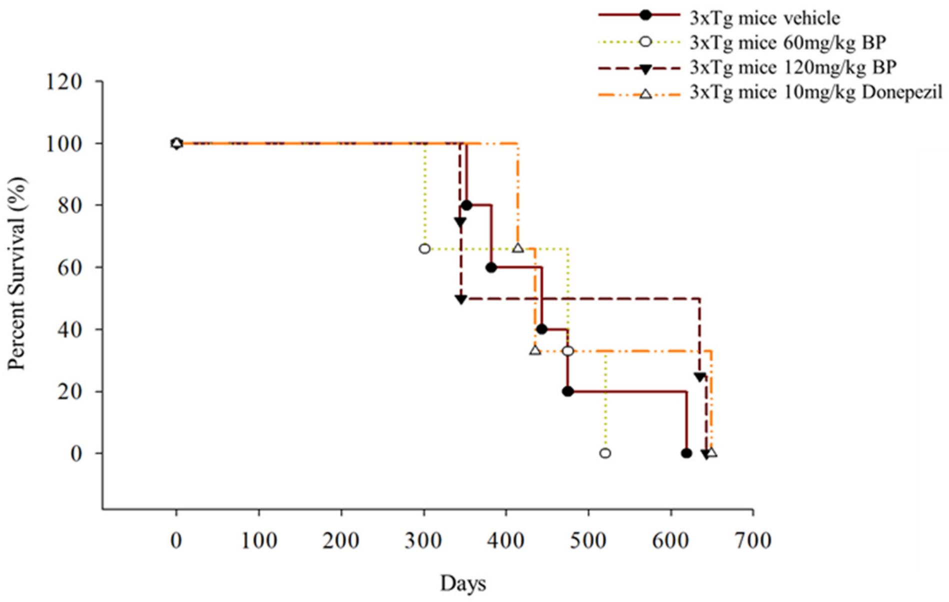

Supplementary Figure S11. Effects of BP treatment on 3xTg mice of survival. Treatment with either oral BP 60 mg/kg or 120 mg/kg with 3xTg mice from 3.5 month old to the natural death, respectively. BP treatment resulted no significant survival shorted as compared to 3xTg mice vehicle(oral oil with 3xTg AD mice) or positive control (oral 10mg/kg Donepezil with 3xTg AD mice). The survival rate of AD Vehicle (454 ± 104 days), n = 5 ; 60mg/kg BP (432 ± 115 days), n= 3 ; 120mg/kg BP (492 ± 170 days), n = 4; 10mg/kg Donepezil (499 ± 130 days), n = 3.

Supplementary Figure S12.

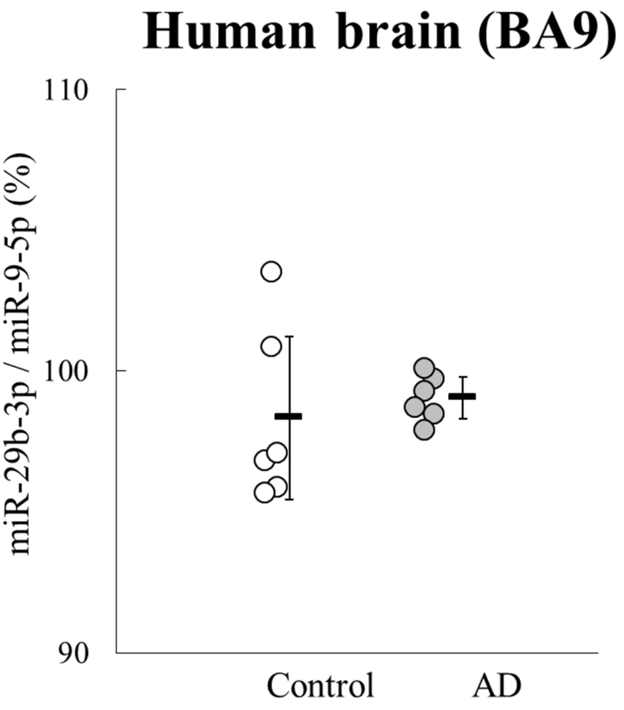

Supplementary Figure S12. The expression levels of miR-29b-3p. The expression level of miR-29b-3p in human brain BA9 specimens as compared to age-matched control patients using RT-qPCR analysis. (Relative percentage of miR-29b-3p/miR-9-5p- Control: 100% ± 3.23 vs. AD: 100.72% ± 0.82).
